# Replication and Explorations of High-Order Epistasis Using a Large Advanced Intercross Line Pedigree

**DOI:** 10.1371/journal.pgen.1002180

**Published:** 2011-07-21

**Authors:** Mats Pettersson, Francois Besnier, Paul B. Siegel, Örjan Carlborg

**Affiliations:** 1 Department of Animal Breeding and Genetics, Swedish University of Agriculture Sciences (SLU), Uppsala, Sweden; 2 Department of Animal and Poultry Sciences, Virginia Polytechnic Institute and State University, Blacksburg, Virginia, United States of America; 3 Department of Cell and Molecular Biology, Uppsala University, Uppsala, Sweden; University of Washington, United States of America

## Abstract

Dissection of the genetic architecture of complex traits persists as a major challenge in biology; despite considerable efforts, much remains unclear including the role and importance of genetic interactions. This study provides empirical evidence for a strong and persistent contribution of both second- and third-order epistatic interactions to long-term selection response for body weight in two divergently selected chicken lines. We earlier reported a network of interacting loci with large effects on body weight in an F_2_ intercross between these high– and low–body weight lines. Here, most pair-wise interactions in the network are replicated in an independent eight-generation advanced intercross line (AIL). The original report showed an important contribution of capacitating epistasis to growth, meaning that the genotype at a hub in the network releases the effects of one or several peripheral loci. After fine-mapping of the loci in the AIL, we show that these interactions were persistent over time. The replication of five of six originally reported epistatic loci, as well as the capacitating epistasis, provides strong empirical evidence that the originally observed epistasis is of biological importance and is a contributor in the genetic architecture of this population. The stability of genetic interaction mechanisms over time indicates a non-transient role of epistasis on phenotypic change. Third-order epistasis was for the first time examined in this study and was shown to make an important contribution to growth, which suggests that the genetic architecture of growth is more complex than can be explained by two-locus interactions only. Our results illustrate the importance of designing studies that facilitate exploration of epistasis in populations for obtaining a comprehensive understanding of the genetics underlying a complex trait.

## Author Summary

This study provides evidence for a strong and persistent contribution of epistatic interactions to the selection response in two chicken lines subjected to 50 generations of divergent selection for 8-week body weight. We show that the genetic architecture of the trait involves genetic interactions of both second- and third-order and that, together, they explain a large portion of the phenotypic divergence between the lines. By replicating a radial epistatic network found in an independent intercross from the same founder individuals, we show that the type of genetic interactions affecting this complex trait is persistent over time. In addition to replicating pair-wise interactions, the size of the pedigree also facilitated evaluation of third-order interactions, which allowed us to further describe the complex genetic mechanisms underlying growth phenotype in chicken. Moreover, a new approach for measuring and detecting capacitating epistasis was proposed. By showing the importance of third-order epistasis in this system, we reinforce the importance of taking it into account when designing experiments aimed at elucidating the genetic architecture of complex traits.

## Introduction

The regulation of most biological traits is complex and results from the action and interaction of multiple genes and environmental factors. Phenotypic variability within populations resulting from polymorphisms in the genes regulating these traits is the key to evolutionary change of the phenotypes over time, resulting from natural or artificial selection (selective breeding), as well as differences in responses of individuals to e.g. environmental challenges leading to disease. Identifying key polymorphisms and how they contribute to trait expression, i.e. dissection of the genetic architecture of the trait, is therefore of considerable interest in fields such as evolution, agriculture and medicine. The availability of affordable, large-scale assays of genome-wide genetic variation has facilitated a large number of studies to elucidate the genetic regulation of complex traits. The focus of studies has generally been to identify the effect of individual polymorphisms on trait expression, and in this way several individual genes and polymorphisms making large contributions to trait expression have been identified [1]. Thus, genetic interactions between multiple factors (epistasis) have been largely ignored. Epistasis has, however, been suggested as an explanation for several unexplained empirical observations in quantitative genetics, including discrepancies between heritability and the sum of the individual effects of the loci shown to influence the traits [2]–[4], ability for populations to respond to selection without a resulting loss in genetic variation [5], difficulty to replicate and clone complex trait loci [6] as well as inability to identify causative mutations for multifactorial disorders [7]. Genetic interactions have also been suggested as a potentially important mechanism in evolutionary genetics, partly due to their effects on fixation rates of alleles under selection, but also on a systems biology level. In particular, adaptability and robustness are often explained in terms of epistasis [8], [9]. Although there are several theoretically possible explanations for the phenomena described above, the potential importance of epistasis is considerable and exploration of the role of interactions in the genetic architecture of complex traits can provide insights for a wide range of fields. To identify and understand the contribution of epistasis in the genetic architecture of complex traits, larger datasets and more involved statistical analyses are required. As a result, relatively few studies have the potential to evaluate the contributions of epistasis to multifactorial trait expression.

### Evidence for epistatic interactions in genomic studies

In genomics, gene interaction networks have been evidenced in numerous biological systems using e.g. expression profiling [10], protein-protein interaction [11] and gene-knockout [12] studies. In genetics, although the theory is established and has been discussed, replicable genetic evidence linking genetic polymorphisms in genetic networks to phenotypic expression has proven difficult to obtain. In this paper, we describe the replication and in-depth exploration of a multi-locus gene-interaction network previously shown to explain nearly half of the long-term selection response in a bi-directional selection experiment in chickens [13]. It was previously shown that this gene-interaction network did not only explain responses to selection, but was also a likely contributor to the smaller than expected decreases in genetic variation in the selected lines [14] as well as the lower than expected power to map individual loci determining body weight (i.e. the selected trait) for which the lines showed an eight-fold phenotypic difference [15]. The earlier results were obtained using data from the original finding of the network in an F_2_ cross between the selected lines [13]. As a result, the resolution of the inferred QTL was limited to confidence intervals covering >10 Mb in each identified locus. This did not allow for discrimination of whether the estimated genetic effect estimates were due to one interacting gene in each of the segments or if they were a composite effect of a larger number of interacting genes.

### Objective of the present study

To replicate and study the QTL inferred in the original population, an eight-generation advanced intercross line (AIL) was bred from the founder individuals of the original F_2_ population [13]. These data were recently used by Besnier *et al* [16] to screen nine selected chromosomal segments for marginal (additive) QTL effects, and ten loci in these segments had significant individual effects on body weight. Six of the nine chromosome regions contained loci involved in the radial network of epistatic QTL reported by [13] and the central locus in that network, *Growth9*, was shown to contain two independent QTL, which were then designated *Growth9.1* and *Growth9.2*.

Here, we used the large AIL pedigree to study whether the original finding of strong epistatic interactions in QTL network replicated in this independent dataset and to explore the network further by extending the analyses to also include three-locus interactions. By replicating, fine mapping and extending the QTL-network in this independent population, we show that the epistasis can be stable across generations, i.e. the estimates of the QTL interactions are similar, and that interactions higher than second order are important in the genetic architecture of the selected trait. The implications of the replication of epistatic interactions across generations and novel insights that can be gained from also studying three-locus interactions are discussed. Suggestions are also given concerning designs of future studies to elucidate the role of epistasis in the genetic architecture of complex traits. A new method for performing genome wide scans for third order interactions is also introduced.

### Concepts of higher-order epistasis—capacitating epistasis and genotype plane variances

**Figure 1:**
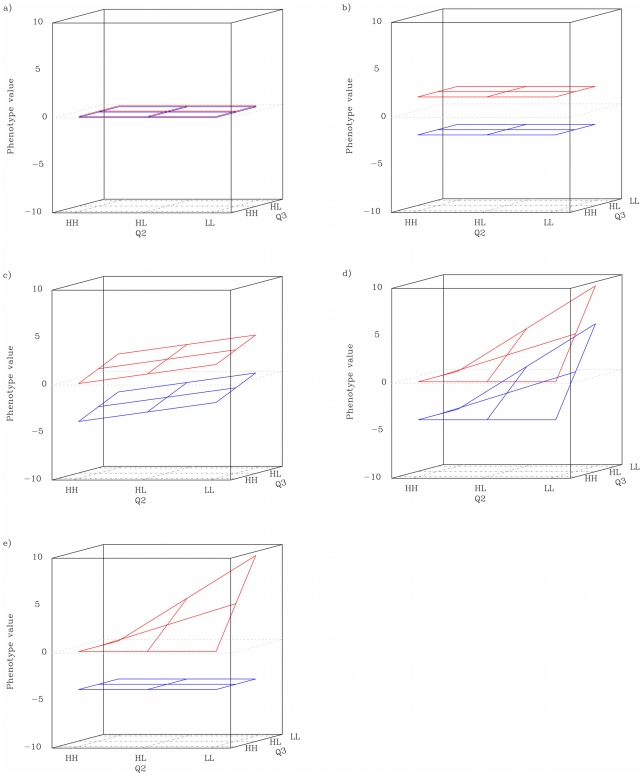
Schematic description of genotype-conditioned plane in different QTL patterns. *a*): neither locus has any effect. *b*): the conditioning locus has a main effect but no interactions. *c*): all three loci have (non-interacting) main effects. *d*): the conditioning locus has a capacitating effect, and the two other loci display synergistic epistasis in the HH background. The red and blue planes represent the HH and LL genotype classes at the conditioning locus, respectively. The phenotype scale is arbitrary.

When a locus acts as a capacitor, its genotype modifies the genetic effects of other loci to be either smaller or larger. For third order epistasis, capacitation can be studied by examining the genotype-phenotype map for triplets of loci, and comparing differences between the “planes” or “slices” of the three dimensional map. Each plane is a two-locus genotype-phenotype map and the effect of the third locus is observed as the distance between the planes for the other two loci (Figure 1). Let us assume a phenotype governed by three loci - Q1, Q2 and Q3 - where Q1 is the conditioning locus. If neither locus has any effect, the planes corresponding to the “HH”, and “LL” genotypes for Q1 should be flat across the genotypes of Q2 and Q3 and also at the same level (Figure 1a). If Q1, and only Q1, has a marginal effect the planes will still be horizontal, with spatial separation due to the marginal effect (Figure 1b). If all three loci have (non-interacting) marginal effects (Figure 1c), or even if Q2 and Q3 interact with each other but not with Q1 (Figure 1d), the planes will no longer be horizontal, but they will have the same shape. If, however, Q1 is interacting with the other two loci, the shape of the planes will differ, and result in them having different within-plane variances (Figure 1e). In particular, if the locus has a capacitating effect, the difference in variance between the planes should be substantial.

By measuring the ratio between the plane with the highest variance and the plane with the lowest variance (henceforth *R_p_*) the capacitating epistatic effect of each locus in a given triplet can be quantified. Calculating this type of measure is computationally efficient compared to regression-based methods, which opens new possibilities for developing more refined strategies for identifying gene-gene interactions. However, scaling and significance testing is not straightforward, as the range of the ratio is strongly affected by the magnitude of the smallest variance. Screening for an effect of loci on the variance rather than the mean appears to have a large potential for identification of interacting loci and the computational efficiency should make exhaustive scans of large data sets feasible in spite of the high dimensionality.

## Results/Discussion

### Identification of epistatic loci in QTL segments

From [16], it was known that the nine segments to be examined in the AIL contained loci with either strong or suggestive evidence of marginal effects on body weight. Here, we aim to explore whether any of these loci also display epistatic effects. Of particular interest was to determine if we could replicate the original radial epistatic network around *Growth9* [13], that was later shown in [16] to contain two separate QTL: *Growth9.1* and *Growth9.2*, was i) also present in the AIL and ii) displayed the same type of capacitating epistasis as in the F_2_ population.

#### Chromosome scans to select markers for further tests for epistasis

Our first step in the analysis was to detect the marker that represents the most likely location of an epistatic QTL in each chromosomal segment. This was done using a two-dimensional scan for interacting loci, where a statistical model including marginal (additive and dominance) as well as epistatic (additive-by-additive, additive-by-dominance, dominance-by-additive and dominance-by-dominance) interactions was fitted for all pairs of markers in the nine segments as in [17]. In each of the nine segments, the marker location showing the strongest support for interactions was selected for further significance testing for its involvement in epistatic interactions with the selected markers in the other segments. The locations of the nine selected markers (one in each segment) are given in Table 1. The complete result of the two-dimensional scan used to select these loci is shown in Figure S1. The original *Growth9* QTL was shown in [16] to contain two linked, independent QTL. The two-dimensional scan (Figure S1) did not indicate two loci with distinct epistatic interactions with loci on other chromosomes. Therefore, we considered only epistatic interactions with one marker on chromosome 7 (close to *Growth9.1*) in the remainder of this report. The genotypes at the markers that displayed the strongest interactions in each chromosome segment (Table 1) were used in the further analyses described below including estimation of multi-locus Genotype-Phenotype maps and estimation of QTL interaction effects.

**Table 1:** Marker locations with the strongest interactions in the nine tested chromosomal segments.

QTL region	Start (Mb)	Stop (Mb)	Selected marker	Position (Mb)
*Growth1*	169.6	181.1	5	174.1
*Growth2*	47.9	65.5	33	61.3
*Growth3*	124.3	133.6	16	132.7
*Growth4*	24.0	68.0	25	33.9
*Growth6*	1.3	13.5	25	13.5
*Growth7*	85.5	88.8	13	88.4
*Growth8*	33.7	39.1	3	36.3
*Growth9*	10.9	35.5	44	21.8
*Growth12*	7.1	13.9	8	9.0

### Replication and explorations of capacitating epistasis in a multi-locus radial QTL network

#### Single-marker analysis to replicate capacitating epistasis

Strong epistasis was previously reported in a radial QTL network with *Growth9* at the center [13]. The epistasis was a capacitating reciprocal interaction with two distinctive features: First, that high-line alleles at the central locus (*Growth9*) released the effects of the three radial loci (*Growth4*, *Growth6* and *Growth12*) and second, that high-line alleles at the radial loci released the effects of the central locus. Consequently, the total effect of the network could be released (or suppressed) by introduction of high-weight (or low-weight for suppression) alleles either at the central locus or at the radial loci. Here, we tested whether this radial network architecture and capacitating reciprocal interaction could be replicated, and potentially also extended, in the AIL pedigree.

#### Estimating contribution of pair-wise QTL effects in the radial network

To evaluate the contribution of capacitating epistasis to body-weight, one needs to estimate the contribution of the loci in a fully capacitated network as well as in a fully suppressed network. Assuming that the main capacitation occurs in the radial network topology around *Growth9* as in the original study [13], the capacitation effect can be estimated by the difference in the additive effects of the radial loci in the *Growth9* high-line (HH) and low-line (LL) homozygote backgrounds and reciprocally the additive effects of *Growth9* in the HH- and LL-backgrounds for the radial loci.

In the AIL, the additive effects differed in HH and LL backgrounds at *Growth9* for five (*Growth2*, *Growth4*, *Growth6*, *Growth8* and *Growth12*) of the eight QTL. They differed either by being significantly higher in the HH genetic background and/or by being significant in one of the genetic backgrounds and non-significant effect in the other (Table 2). Four of the five loci that interacted with *Growth9* (*Growth2*, *Growth4*, *Growth6* and *Growth12*) were part of the original epistatic QTL network detected in [13]. The sum of their effects was 4.7-times higher in a HH than LL genetic background at *Growth9* (Table 2) and explained approximately 30% of the difference in body weight between the HWS and LWS lines. The fifth locus that interacted with *Growth9*, *Growth8*, was not part of the original network [13]. In the AIL, *Growth8* had a strong transgressive epistatic effect, i.e. the HWS alleles at *Growth8* increase body weight in a LL genetic background at *Growth9*. The locus did not have a detectable effect in a HH background (Table 2). A re-analysis of the data from the original F_2_-cross shows that this transgressive effect can also be observed in that population, although it did not reach genome-wide significance in the original analysis. In total, the estimated effect of the epistatic network is somewhat smaller in the AIL than in the original study (about 30% vs 45% of the parental line difference or, if measured as number of phenotypic standard deviations in the analyzed pedigrees, 1.9 vs 2.2 σ_P_). The decrease in effect is thus much larger (33%) when compared against the parental line difference than when compared to the available variation in the analyzed pedigree (13%). This is a result of the small absolute size, and thus variation, of the F_8_ generation, which constitutes a large part of the pedigree; the standard deviation of the F_8_ generation is 127 g, compared to 187 g in the original F_2_ cross.

**Table 2:** Genetic effects of five epistatic QTLs for body weight in the Viriginia AIL in alternate genetic backgrounds.

TestedQTL	StratificationQTL	a ± SE (g)HH	a ± SE (g)LL	Pa (HH)>a (LL)
**Additive**				
*Growth2*	*Growth9*	30.4±17.9	−34.7±16.9	0.016
*Growth4*	*Growth9*	62.0±17.0	22.7±17.3	0.048
*Growth6*	*Growth9*	35.4±17.6	4.3±18.8	0.096
*Growth9*	*Growth2*	90.2±18.8	21.7±17.4	0.011
*Growth9*	*Growth4*	60.0±17.3	21.4±16.1	0.046
*Growth9*	*Growth6*	39.4±22.9	9.0±17.2	0.103
*Growth12*	*Growth9*	41.4±16.7	19.1±15.7	0.195
*Growth9*	*Growth12*	45.5±16.0	22.0±15.6	0.143
**Sum A:** **% population difference**		404.330%	85.56%	6%
**Transgressive**				**P** **a (HH)<a (LL)**
*Growth8*	*Growth9*	11.4±18.9	54.1±20.0	0.055
*Growth9*	*Growth8*	7.9±21.7	50.8±17.4	0.055
Sum A:	*Growth9*	19.3	104.9	
*Growth9*	*Growth8*	7.9±21.7	50.8±17.4	0.055
Sum A:		19.3	104.9	

Estimated additive effects with standard errors (a±SE) are given for each QTL in strata containing only individuals homozygous for HWS (HH) or LWS (LL) derived alleles at the Stratification QTL. The QTL where the HWS alleles increase the body-weight in the HH/LL strata respectively are given under the headings Additive/Transgressive (see text for more explanation).

#### Analysis to evaluate robustness of epistatic interactions

**Figure 2:**
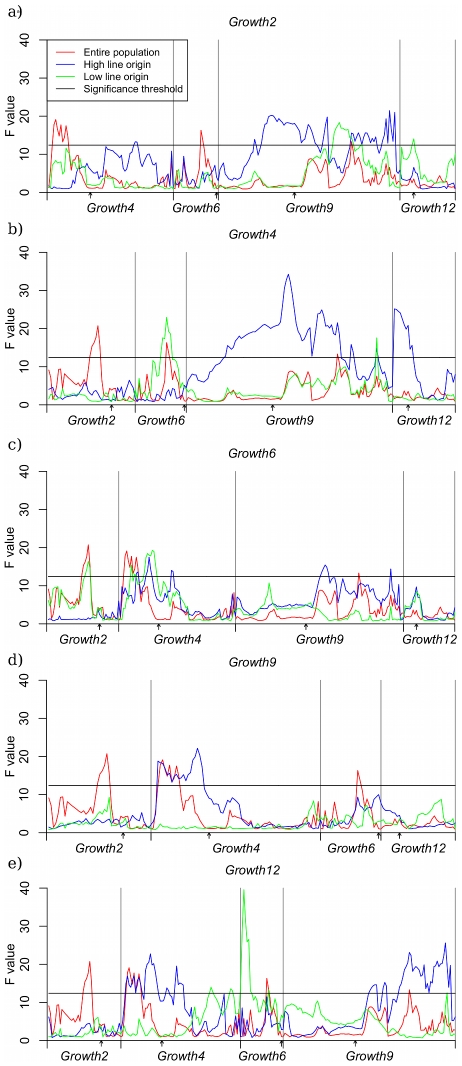
QTL scans on conditioned subsets of data. Panel *a* contains the scan where *Growth2* is the conditioning locus, in *b*, *c*, *d* and *e*, the conditioning loci are *Growth4*, *Growth6*, *Growth9* and *Growth12*, respectively. Each panel shows the QTL profile in three data sets: HWS background (blue), LWS background (green) and the entire pedigree (red). Scans were performed for the non-transgressive loci in the radial network. The significance threshold represents 95% study-wide confidence based on a permutation test using 1000 replicates.

The regions that displayed capacitating epistasis in the single marker analysis described above were subjected to further analysis to evaluate the robustness of the pair-wise interactions in the radial epistatic network. For this analysis, one-dimensional QTL scans were performed in all the HH- and LL- stratified data sets described in the Materials and Methods section. In this analysis the chromosomal segments containing *Growth2*, *Growth4*, *Growth6* and *Growth12* were screened using individuals that were high-line homozygous for *Growth9* (HH-*Growth9* strata) and then screened again using individuals that were low-line homozygous for *Growth9* (LL-*Growth9* strata), and so on until all segments had been screened for all data. The results of these scans are shown as bootstrap-averages in Figure 2.

For the datasets conditioned on the radial loci (*Growth2*, *Growth4*, *Growth6* and *Growth12*), only the HH stratum (except a single maker in the *Growt6* LL stratum) showed a QTL signal above the significance threshold across the *Growth9* region (including both *9.1* and *9.2*) (Figure 2). *Growth4* and *Growth9* exhibited a significant reciprocal interaction as in the original study. In the AIL, *Growth2*, *Growth4* and *Growth12* displayed a unidirectional capacitation of *Growth9*. Although these results thus imply a replicable interaction effect between these loci that is stable across the generations, the capacitation effect of *Growth9* is not as pronounced as in the original study where it capacitated all radial loci. In the analysis we also evaluated interactions between the radial loci in the network. The results show that all the radial loci also interacted with at least one other radial locus, indicating a more complex network topology not limited to the originally evidenced pair-wise radial network. To shed more light on this more complex interaction network, we evaluated the potential impact of higher order interactions on phenotypic expression. The first step in understanding the contribution of the network is to estimate the contribution of pair-wise interactions to the phenotype in a way resembling that described in the F_2_ population [13].

#### Evaluation of multi-locus network effects using a non-parametric genotype-phenotype map

**Figure 3:**
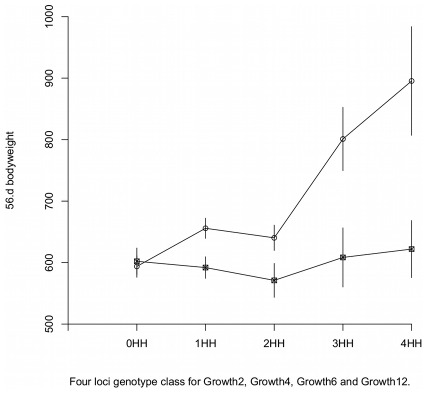
Genotype-phenotype maps in HWS and LWS genetic background at *Growth9.1*. Model free estimation of phenotypic values. The values are plotted as a function of the degree of HWS and LWS homozygosity at its interacting loci *Growth2*, *Growth4*, *Growth6* and *Growth12*. The error bars represent s.e.m. Circles are Growth9 HH and squares are Growth9 LL.

To examine the effects of higher order interactions, we estimated a partial, model-free, genotype-phenotype map using the observed phenotypic mean for each genotype. Here, the phenotypic means were calculated for a subset of genotype classes to which the studied individuals were assigned. Individuals were classified based on the number of HH genotypes they carried in the four non-transgressive radial loci (*Growth2*, *Growth4*, *Growth6*, and *Growth12*); that is, the individuals were assigned to one of five groups depending on whether they were HWS homozygous for 0, 1, 2, 3 or 4 of the radial loci. From these five groups, individuals that were HWS or LWS homozygous at *Growth9* were separated to obtain in total 10 genotype-classes for which sex and generation corrected phenotypic means were calculated. The results, presented in Figure 3, show that i) the 5 locus HWS and 5 locus LWS homozygote genotypes for *Growth2*, *Growth4*, *Growth6*, *Growth9* and *Growth12* gave the highest and lowest phenotype values, respectively; ii) Only in the *Growth9* HH homozygous genotypic background did the predicted phenotype increase with the addition of HWS alleles at the radial loci.

The results from the predicted multi-locus GP-map is thus in agreement with the results from analyses of the stratified data in that LWS homozygosity at *Growth9* inhibits the growth-promoting effects of HWS alleles in *Growth2*, *Growth4*, *Growth6* and *Growth12*, while HWS homozygosity at *Growth9* releases growth promoting effect at the same loci. Data also indicates that the interaction in the radial network is reciprocal in this analysis, which is consistent with the original observation in the F_2_ population. The HWS allele in *Growth9* had indeed lower growth promoting effects in the LWS than HWS homozygote background of *Growth2*, *Growth4*, *Growth6* and *Growth12*. In addition, we constructed a parametric version of the genotype-phenotype map using the Natural and Orthogonal InterAction (NOIA) framwork [18], which showed similar results (see Text S1 and Figure S2). Figure 2 also shows that the additive effect of *Growth9*, which can be detected in a QTL scan for marginal effects, is caused by an individual genetic effect of *Growth9* in combination with capacitation by beneficial alleles at the four radial loci. The same holds for the marginal additive effects of *Growth2*, *Growth4* and *Growth12*, which also supports the original observation from [13].

### Beyond the radial network—exploring the effects of all detected QTL interactions

To move beyond the observations related to the original network and understand the combined effect of all the identified interacting QTL, we examined the phenotypic values for individuals with a given set of genotypes for all possible pairs and triplets of loci within a set of six loci; the radial network (*Growth2*, *Growth4*, *Growth6*, *Growth9* and *Growth12*) and the QTL *Growth1*. *Growth1* was included because it had a strong individual marginal effect, displayed suggestive interactions in the exhaustive 2D-scan (Figure S1), and had significant interaction effects with four of the other loci when used in a stratified scan of the same type as described for the other loci above (data not shown).

#### Pair-wise epistasis

**Figure 4:**
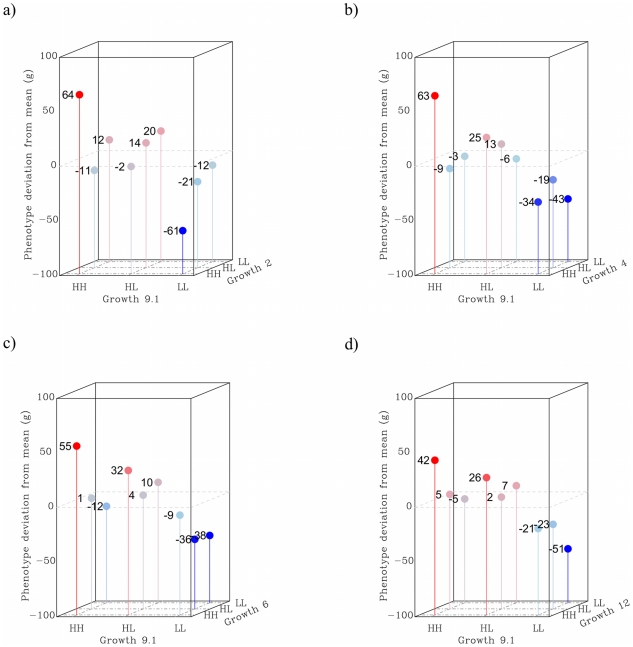
Two-way interactions in the radial QTL network. Panel *a* shows the interaction between *Growth9.1* and *Growth2*, where *Growth2* on average has virtually no effect in this pedigree. However, stratification reveals that it has an effect, in opposite directions, in both the HH and LL background at *Growth9.1*. The remaining panels show the synergistic interactions between HH-alleles in the three pairings between *Growth9.1* and *Growth4* (*b*), *Growth6* (*c*) and *Growth12* (*d*) in turn.

The results for the locus pairs are presented in Figure 4, where shown are sex- and generation corrected phenotypic means for all two-locus genotypes. To correct for fixed effects, we compared the mean of each individual to the subset of the pedigree that was of the same sex and belonged to the same generation. The values presented are individual deviations from the mean phenotype of the class (i.e. same sex and generation) and are centered on zero. From these results we can conclude that capacitating interactions are more than just a feature of the radial QTL network. This is because most evaluated locus pairs involved some degree of capacitating epistasis, where the HH-genotype at *Growth9* increased the effect of the other locus. An exception to this is *Growth2*, because it appears to be a strong capacitating locus with virtually no individual marginal effect; all its influence on the phenotype was due to interactions.

#### Higher-order epistasis

If a complex trait determined by *n* loci is considered, the phenotype of each individual in a population will be the result of its individual *n*-locus genotype acting in its internal and external environments. In our studies, we were limited by neither knowing the true number of loci (*n*) affecting growth nor how inter-dependent the genetic effects of these loci were in determining growth. Therefore, in our genetic analyses we first sought to detect loci determining the trait and then estimate their effects by calculating phenotypic means for *k*-locus genotypes (*k*> = 1). Although technically the upper bound is *k* = *n*, very high order effects are not likely for mechanistic reasons and difficult to detect due to the rarity of the individual genotypes for high *n*. When *k*<*n*, the phenotypic means will be an average across the genotypes of all the remaining (*n-k*) loci that affect the trait. If there are no interactions between the *k* primary loci and the remaining (*n-k*) loci, these analyses will provide estimates of genetic effects that sum to the total genetic effects on the trait. If there are interactions, however, they will not. To explore how results are affected by taking averages over different subsets of loci, we estimated the phenotypic means for the detected QTL using *k = 1*, *k = 2* and *k = 3* (accurate estimates for higher values of *k* cannot be obtained due to too few observations). Table 3 contains results for one subset of three loci (*Growth4*, *Growth9* and *Growth12*). The effect of considering multi-locus genotypes for phenotypic predictions is shown by the increase of explained variation with higher values of *k*.

**Table 3:** Differences in total effects of the three loci *Growth4*, *Growth9.1*, and *Growth 12* based on their context.

	*Growth4*	*Growth9*	*Growth12*	Total (g)
*k* = 1	33	43	28	104
*k* = 2	(106/2+63/2)/2 = 42	(106/2+93/2)/2 = 50	(63/2+93/2)/2 = 39	131
*k* = 3	207/3 = 69	207/3 = 69	207/3 = 69	207

The rows show the effect of the triplet when increasing numbers of loci are taken into account. The first row (*k* = 1; only the primary locus) gives the summation of the marginal effects of the three loci. The second row (*k* = 2; primary locus with one background locus) gives the effect the loci calculated as the average over the two pairs that it is part of (each locus is assumed to contribute half the effect of the pair). The third row (*k* = 3; all three loci together) gives the effect for each locus is a third of the entire triplet effect. As can be seen, using either *k* = 1 or *k* = 2 leads to underestimation of the total impact of the three loci.

**Figure 5:**
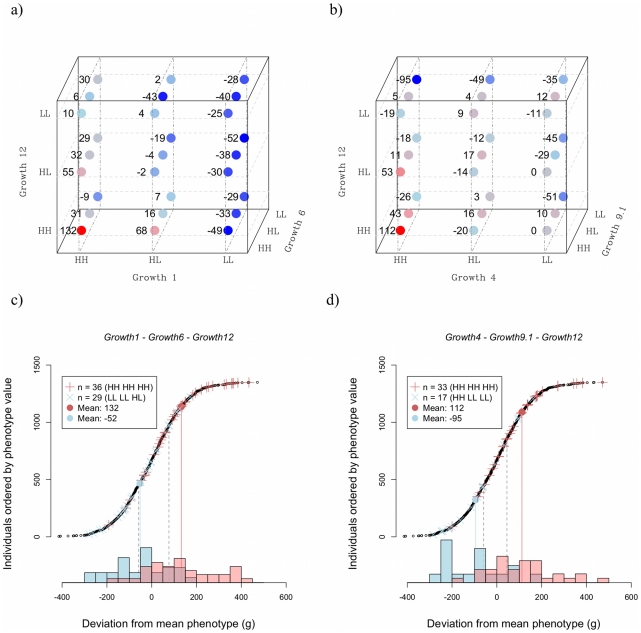
Three-way epistatic interactions. Panel *a* shows the mean phenotype values for each genotype for the triplet *Growth1*-*Growth6*-*Growth12*; panel *b* shows the same for the triplet *Growth4*-*Growth9.1*-*Growth12*. The values shown are the mean (over all individuals with that genotype) of the difference from the average for the class – that is all individuals with the same sex and belonging to the same generation. Thus, positive values in the graph indicate that individuals carrying that genotype are, on average, larger than expected for their class. Panel *c* shows the distribution of phenotype values for the *Growth1*-*Growth6*-*Growth12* triplet, and panel *d* shows the same for the *Growth4*-*Growth9.1*-*Growth12* triplet. The red “+” symbols show individuals with the high-weight genotype, the blue “x”-symbols indicate individuals with the low weight genotype. The correspondingly coloured circles show the genotype mean. Phenotypic values for all individuals are shown as black circles and the inserted histograms show the phenotype distributions for the high-weight (red) and low-weight (blue) genotypes. The expected range based on the additive effects of the loci is shown as dashed grey lines.

Figure 5 shows how higher order epistasis contributes to phenotypic expression for two triplets of loci (*a*, *c*: *Growth1*-*Growth6*-*Growth12*; *b*, *d*: *Growth4*-*Growth9*-*Growth12*). In the *Growth4*-*Growth9*-*Growth12* triplet, the difference in weight between the genotype with the highest phenotypic value (HH-HH-HH) and that with the lowest (HH-LL-LL) is 207 g. This number nearly doubles the expectation from the summation of the individual effects of the loci (Table 3).

The capacitating effect of *Growth1* is illustrated in the results presented in Figure 5*a*. Here, the LL genotype at *Growth1* suppresses the effect of *Growth6* and *Growth12*. As a reference, the *R_p_*-value for *Growth1* in this triplet is 18, which is the highest among the possible three-locus combinations of the six loci (Figure S3), thus, in this picture, *Growth1* displays the strongest capacitating effect of the involved loci. In addition, *Growth6* and *Growth12* are strongly affected by the genetic background at *Growth4*; in this triplet the *R_p_* value is 16. Figure 5 also illustrates the joint effects of the QTL triplets (*Growth1*-*Growth6*-*Growth12*; Figure 5c) and (*Growth4*-*Growth6*-*Growth12*; Figure 5d). Here, the residual phenotypic distributions of individuals carrying either the best or worst three-locus genotypes for these triplets are plotted together with the additive expectations, illustrating the strong capacitating effect of the interactions. For the *Growth1*-*Growth6*-*Growth12* triplet, the deviation from additivity is mainly due to the phenotype in the high-weight genotype, which is unexpectedly large. For the *Growth4*-*Growth9*-*Growth12* triplet, the interaction elevates the phenotype in the high-weight genotype and suppresses the phenotype in the low-weight genotype. The phenotypic distribution of the individuals in the pedigree is also shown, to provide a point of reference as to the scale of the measure. Figure 5 thus provides a model-free visual measure of the total effects of the triplets, and also a comparison with the phenotypic distribution of the entire pedigree. The genotypes fall into distinct distributions, with larger mean differences than would be expected from summing the marginal effects, or the pair-wise interactions, which supports the conclusion that three-locus interactions are important for body-weight in this system. However, due to the large number of estimated, and thus uncertain, values that need to be combined to calculate these numbers, it is not possible to state that this observation is statistically significant; that would require a data set at least one order of magnitude bigger than the, already large, one used for this study.

The analyses reported in this paper all demonstrate a complicated genetic architecture of the selected trait. The original radial network of pair-wise epistasis, evidenced in the original report [13], is in this more thorough investigation shown to be a part of an even more complex structure. An additional important locus, *Growth1*, is identified and new, higher order, interactions among the previously identified loci are shown to make large contributions to body-weight. While the molecular mechanisms are still unknown, the results suggest that some of the involved loci are upstream regulators of the key metabolic pathways involved in growth. *Growth1*, which has been linked to many associated phenotypes [19], [20] and show strong capacitating effects in this study, is a likely candidate to in the same way as *Growth9*, act as a key capacitor locus in the complete network.

#### Implications of capacitating epistasis for complex trait genetics

In this study we identified abundant capacitating epistasis in a network of loci important for the large selection response in the body-weight selected Virginia chicken lines. As capacitation has released selectable genetic variation in the HWS line and suppressed genetic variation in the LWS line, it has made it possible to obtain a response to selection far outside the range of phenotypes in the common base-population. This change in the total selectable additive genetic variance results from a release of cryptic (or standing) genetic variation, which has earlier been implicated as a source of Selection Induced Genetic Variation (SIGV) [21]; something that was experimentally validated initially [13] in these lines. Being able to estimate the level of capacitating epistasis would also be useful for the ability to predict a population's potential for genetic change in selection programs or adaption to new environments in an evolutionary time scale [14].

The cryptic genetic variation that this epistatic mechanism can hide in a population also has implications for estimation of the heritability in that population, because the marginal genetic (additive) effects of individual loci in a network involving capacitating epistasis will be highly dependent on the allele-frequencies at the other loci in the network. This is clearly illustrated by the network studied here, where the sum of the marginal genetic effects is much higher in a HWS genetic background (i.e. a capacitated network), than in a LWS genetic background (i.e. a suppressed network). The genetic potential [14] - the allele-frequency independent ability of existing alleles in a population to change the phenotype - of the population is thus generally higher than the estimate of the heritability, which is dependent on allele frequencies, in a population where capacitation exist. As a consequence, the sum of the individual marginal effects of the loci detected for a trait may be biased due to capacitating epistasis.

Replication and cloning of complex trait loci becomes more of challenge for loci in capacitation networks. As the effects of the loci are context dependent, it is not possible to use traditional approaches to identify single, main loci and then replicate and clone these independently using standard methods. Instead, it becomes necessary to first identify the interacting loci, which in most cases is difficult due to the costs and work involved in generating sufficiently large datasets, and then to thoroughly identify the dependencies between the interacting loci. Only once that is done, is it possible to develop an appropriate strategy for simultaneous replication and cloning of the key loci. Due to the complexities and the cost involved, we can expect that the ability to identify and clone causative mutations using top-down approaches will impede progress in identifying new loci for multifactorial disorders where capacitation is important.

The potential impact of capacitating genetic interactions in evolution is considerable. The existence of key loci that suppress and release cryptic variation in response to selective forces will allow populations to either stably maintain a robust phenotype while adapted to an environment (a suppressed network displaying little of the genetic potential of the population), or facilitate a rapid phenotypic change in response to changing environments by releasing the selectable genetic variation by capacitating the genetic network through the master regulator loci. It will therefore be of great interest to in a directed way search for capacitating epistasis in evolutionary studies using the *R_P_* measure suggested here, or variations thereof.

### Conclusion

Using a large multigenerational pedigree we are able to demonstrate replication of a multi-locus QTL interaction network in vertebrates. Our results show that the type of interactions detected in the original population replicate to a considerable extent, both regarding the loci included as well as their combined effects. Due to the large population size we were also able to include analyses of higher order interactions that show that the primary interaction mechanism, genetic capacitation, is a main feature of the network that involves not only pairs but also triplets of loci. Based on these results we introduce the idea that high order epistasis can be studied by examining the variance differences between genotypes in multi-dimensional genotype-phenotype maps.

This study provides further evidence for the importance of genetic interactions in determining complex phenotypes and indicates that the value of epistatic analyses in studies aiming at genetic dissection of the architecture of complex traits.

## Materials and Methods

### Animals

An eight generation Advanced intercross line (AIL) was produced from two selected lines of chickens obtained by bi-directional, single trait selection for bodyweight at 56 days of age (referred to as the High Weight Selected “HWS” and Low Weight Selected “LWS” lines). The lines originate from a common base population, consisting of crosses of seven partially inbred lines of White Plymouth Rock chickens [22], [23]. All procedures involving animals used in this experiment were carried out in accordance with the Virginia Tech Animal Care Committee animal use protocols.

Individuals from generation 40 of the HWS and LWS lines were used as founders for the AIL. The sex-averaged 56-day body weight at this generation was 1522 g (SE: ±36 g) for the HWS line and 181 g (SE: ±5 g) for the LWS line. The observed mean heterozygosity, H_0_, at all autosomal loci was calculated as 0.146 and 0.156 in the high and low lines, respectively [24]. The husbandry of the intercross was identical to that of [19]. To produce 100 F_1_ progeny, 10 HWS males were mated with 22 LWS females and 8 LWS males were mated to 19 HWS females). About 100 individuals were produced in generations F_2_, F_4_, F_5_, F_6_ and F_7_ and 300 and 400 individuals in generation F_3_ and F_8_ respectively. The F_8_ generation has undergone the most number of recombination events and should therefore give the best resolution, which is why it is also the largest. The f3 generations was increased in number in order to provide power to detect variation that might be lost deeper in the pedigree.

### DNA extraction, marker selection, and genotyping

Nine chromosome regions with significant or suggestive QTL for body weight in the F_2_ generation [13], [15], [20] were selected for further study in the AIL. The segments are abbreviated as in [15]. For all individuals in the AIL, DNA was extracted from blood by AGOWA GmbH (Berlin, Germany). In addition, 15 individuals from each parental line were genotyped for approximately 13,000 SNP markers, distributed genome-wide, as described in [15]. The nine QTL regions include 384 segregating SNPs, selected out of the 13,000 total SNPs. The average distance between the markers was less than 1 cM. All individuals in the AIL (*n* = 1529) were genotyped for the set of 384 markers using the GoldenGate assay (Illumina, CA) at the SNP technology platform in Uppsala (Sweden).

### Replication of epistatic QTL

To study the effects of the radial network we used the stratification based QTL interaction analysis employed in [13]. A brief description of the procedure is provided below. First, QTL genotype contrasts (regression coefficients that are essentially re-scaled probabilities [25]) were estimated at each marker in the 10 genomic regions evaluated. These contrasts, calculated from a gametic IBD matrix [26]–[29], were used in a two-dimensional scan for interacting QTL pairs where tests were performed using a two-locus epistatic model [17]. Several types (single QTL at each locus, two non-interacting QTL and two interacting QTL) of models were fitted and the loci were considered to be interacting when the interacting QTL model had a significant improvement over the others. Significance was determined from a permutation test procedure, as described in [17]. Based on this analysis, we selected the marker location in each segment that showed the strongest support for interactions as the locus to be tested for significant epistasis.

We generated a series of stratified datasets, i.e. subsets of the data that contained only the individual most likely to be homozygous at a conditioning locus. Two strata for each conditioning locus (one stratum with the LL homozygotes and one with the HH homozygotes) was produced using the genotypic contrasts at the marker position in each selected chromosomal segment that had the strongest indication of epistasis in the two-dimensional QTL scan. We stratified using six conditioning loci (*Growth1*, *Growth2*, *Growth4*, *Growth6*, *Growth9* and *Growth12*), yielding a total of 12 subsets. The sizes of the strata varied between 229 and 377 individuals. These stratified datasets were then used for the interaction analyses.

For pairs of loci where significant interactions were detected, the additive genetic effects of the QTL were estimated separately in each of the three strata. The estimates of the additive effects were obtained using linear regression of residual body weight at 56 days of age, corrected for the fixed effect of generation and sex, as in [13].

The robustness of potential interactions was evaluated using a combined bootstrap and permutation testing strategy, designed to control the type-I errors in this advanced intercross pedigree [30]. For each locus tested, the same analyses were performed for the whole data set, as well as for the HH and LL strata separately. In each dataset, 200 bootstrap samples were generated and analyzed using a one-dimensional QTL scan [31]. For each marker, an additive model was fitted, with sex and generation as fixed effects, the results averaged over all bootstrap replicates. Bootstrap data sets were sampled with replacement and were of the same size as the original data set. The significance threshold was determined by a permutation test, where 1000 datasets were generated by permuting the genotypes of the individuals, while preserving the relationship between phenotype and the two fixed factors, sex and generation in the pedigree. The threshold was selected so that it was above the maximal value found in 95% of the permutations. One-dimensional scans, following the procedure described above, were performed in each dataset, and the distribution of the maximal F-scores from each replicate was used to determine an empirical significance threshold.

Loci defined as epistatic in the two-dimensional scan were included in a joint network analysis as follows: initially, all marginal and two-way interactions for epistatic loci in the network were estimated jointly using the NOIA framework [18]. The use of the “statistical model” in NOIA provided an orthogonal model for the estimation of genetic effects, even though the population did not conform to an ideal F_2_ allelic frequency distribution at each locus. From the orthogonal estimates, a multi-locus genotype-phenotype (GP) map was constructed using the “transformation” operation in NOIA [18]. This GP-map provides estimates of the genotype values (expected phenotypes) for all multi-locus genotype combinations in the network that are useful for functional studies of epistatic interactions.

In addition, phenotypic means of genotype classes were estimated directly using a discretized version of the data set. Genotypes of each individual were discretized at the marker positions based on the genotype contrast value. The contrasts ranged from −1 to 1, with 0.4/−0.4 used as thresholds for assigning a HH, HL, or LL genotype to a marker. These thresholds are conservative (the HL interval is larger than that of the homozygotes; 0.33/−033 would be most natural and split the space evenly), to reduce the number of falsely assigned homozygotes because they are most critical to the outcome. However, because most individuals had accurately estimated genotype probabilities, i. e. contrast values close to −1, 0 or 1, the choice of cut off has little effect on the outcome. We also used the discretized data in a second order interaction model-based NOIA-approach, and constructed a partial GP map directly from the phenotypic means of individuals with given genotypes. The observed means of the genotype classes were then compared to the expected ones from the NOIA model. Differences between these values are an indication of the effect of higher order interactions.

Discretized data were used to study three-way interactions. For a set of triplets of loci, the phenotypic mean (again corrected for sex and generation) for each three-locus genotype was calculated. Thus, for each triplet of loci, means were calculated for 27 classes of individuals, each corresponding to a three-locus genotype. Since each genotype was scored separately, any type of interaction was detected from the pattern of these values. For detection of capacitating epistasis, the 27 means were grouped into three “planes,” where each plane consists of the nine genotypes that shared a single genotype of the potentially capacitating locus (i.e. there is one plane each for the “HH,” “HL” and “LL” genotypes at that locus). Then, the variance within each plane was calculated together with the ratio between the planes with highest and lowest variance. This ratio measures the capacitating effect of the conditioning locus.

## Supporting Information

Figure S1 Complete 2D scan. The profile of an exhaustive 2D QTL scan over all markers in the data set.
(PDF)

Figure S2 Parametric genotype-phenotype maps in HWS and LWS genetic background at *Growth9.1*. The phenotypic values were predicted using the NOIA model framework. The values are plotted as a function of the degree of HWS and LWS homozygosity at its interacting loci *Growth2*, *Growth4*, *Growth6* and *Growth12*. The error bars represent s.e.m.
(TIFF)

Figure S3 Distribution of *R_p_*-values for all possible loci combinations. Panel *a* shows all values, in decreasing order, from all possible combinations within the six loci set. (60 values; 3 for each of 20 possible triplets). Panel *b* shows a histogram of these values.
(TIFF)

Text S1 Prediction of multi-locus network effects using a parametric genotype-phenotype map.
(DOC)
