# Supplementary figures and images for: Replication and Explorations of High-Order Epistasis Using a Large Advanced Intercross Line Pedigree
Source: PLoS Genet. 2011 Jul 21;7(7):e1002180. doi: 10.1371/journal.pgen.1002180 (PMC3140984; doi:10.1371/journal.pgen.1002180)

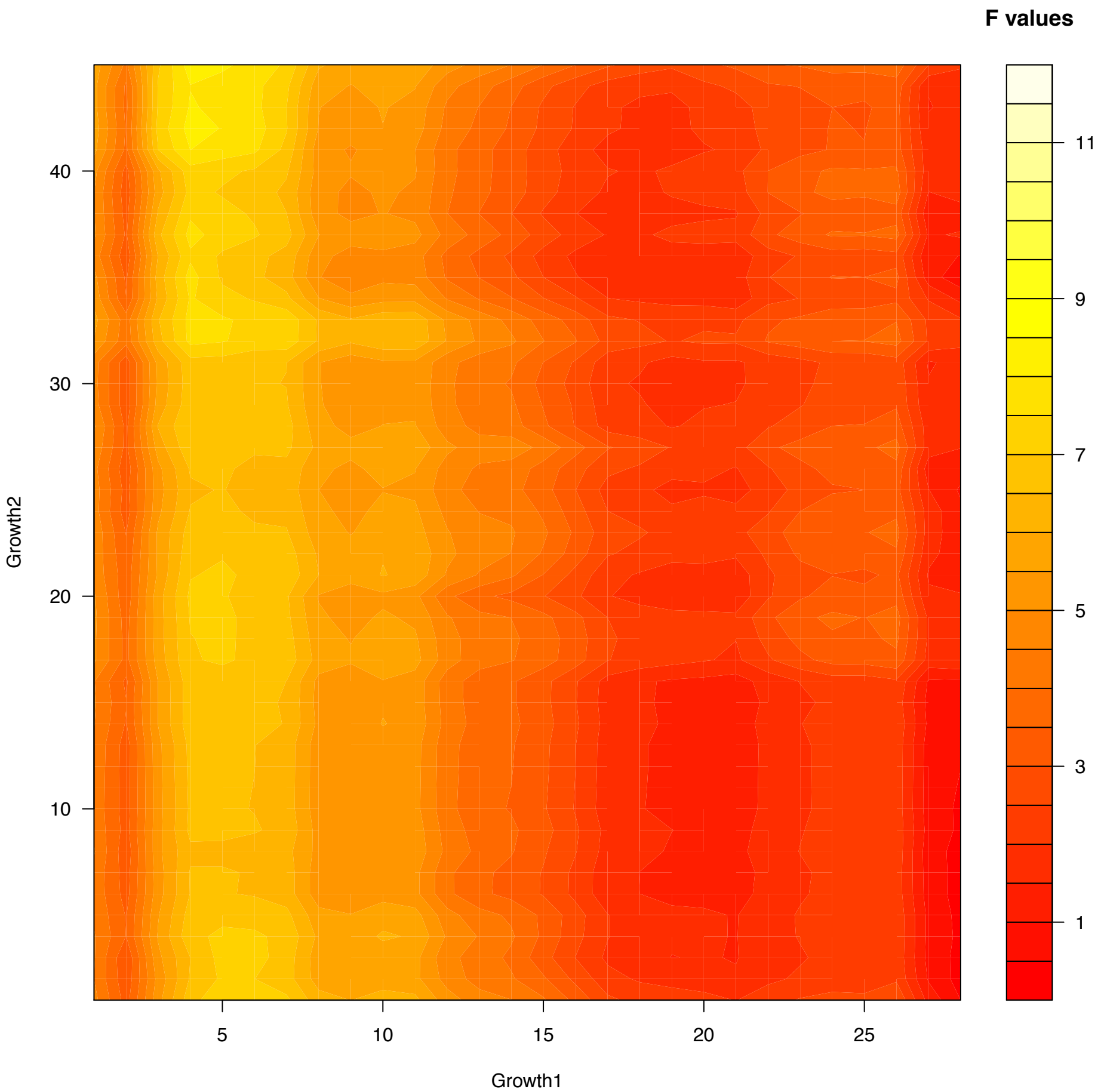

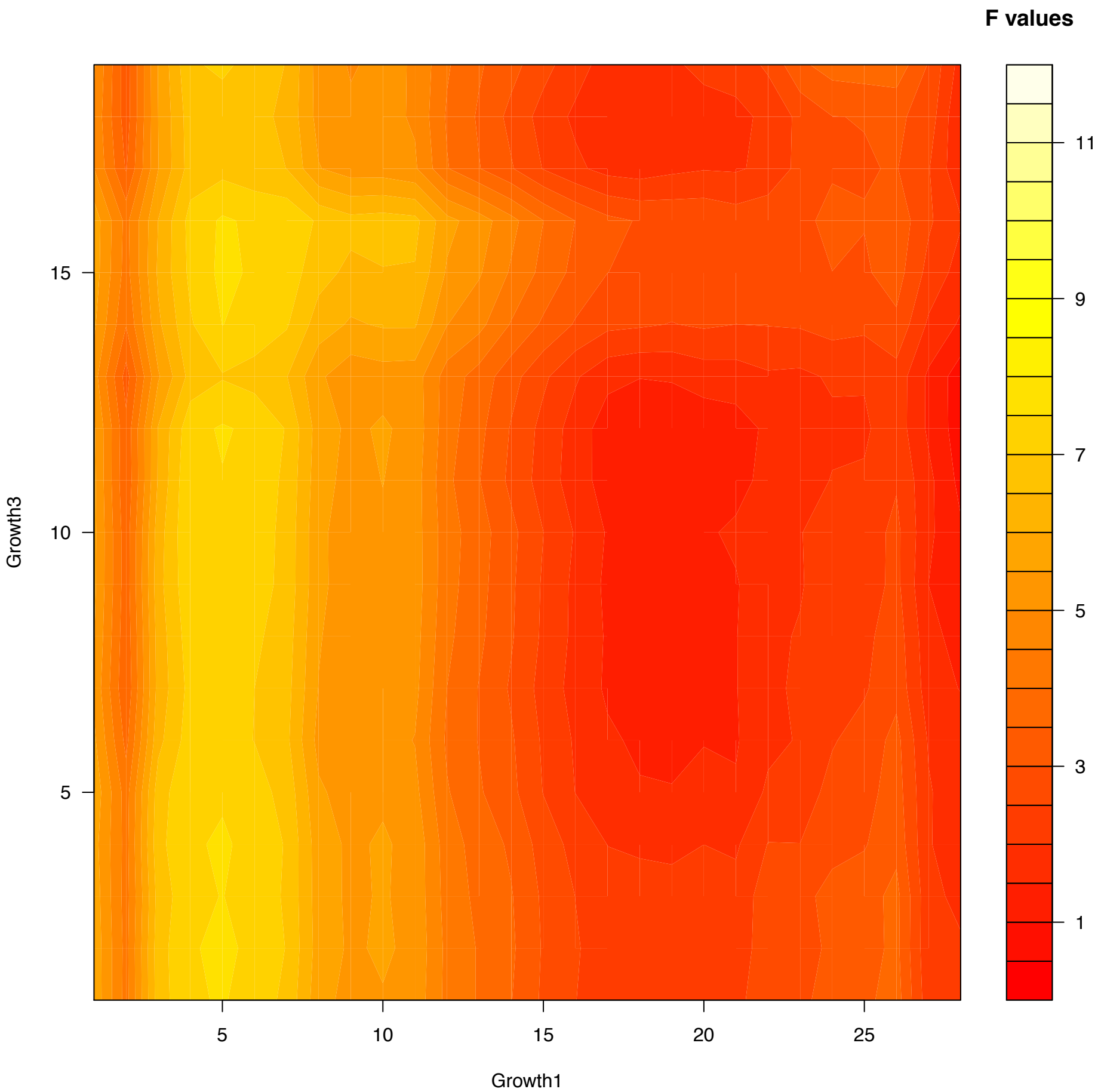

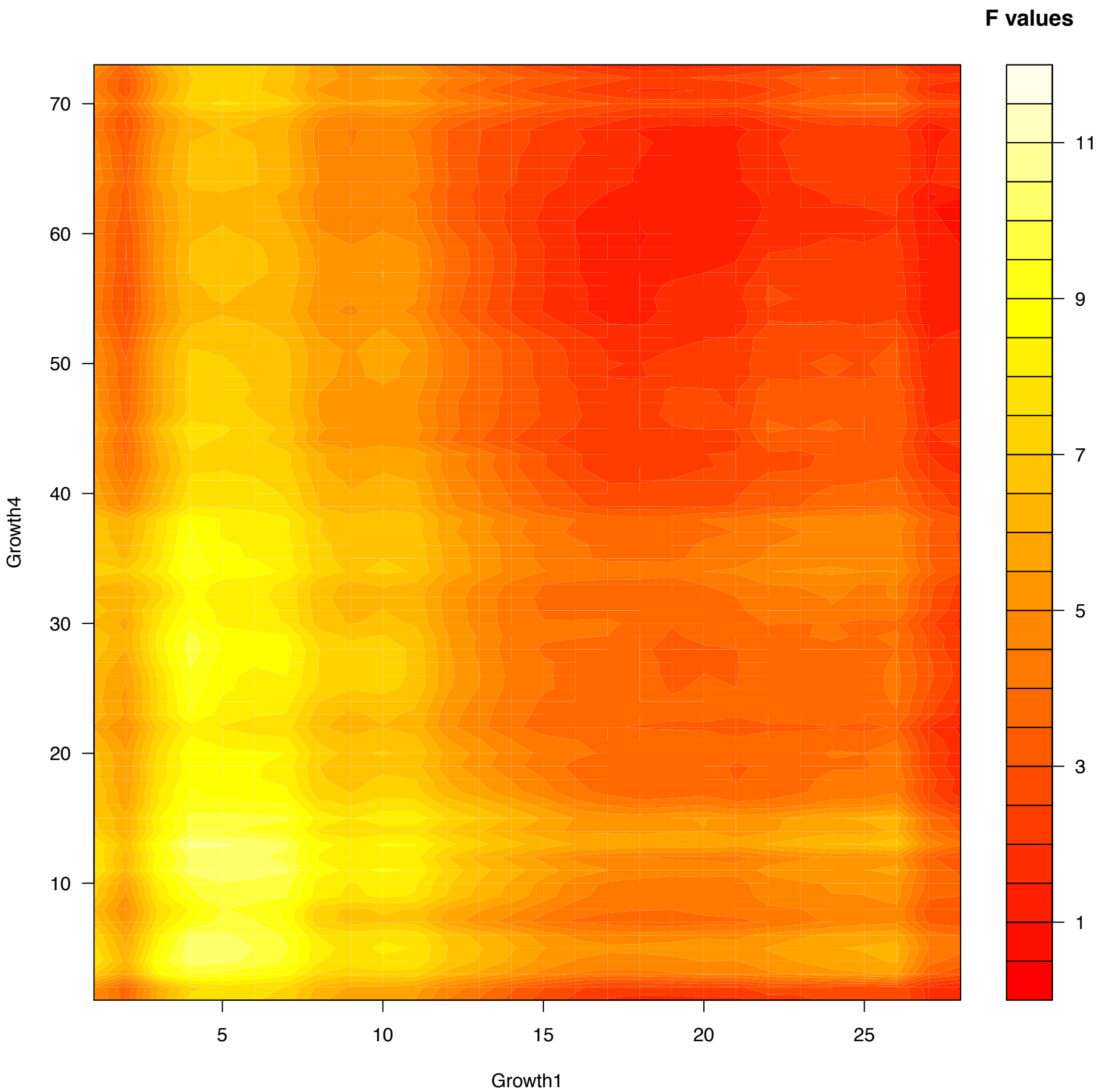

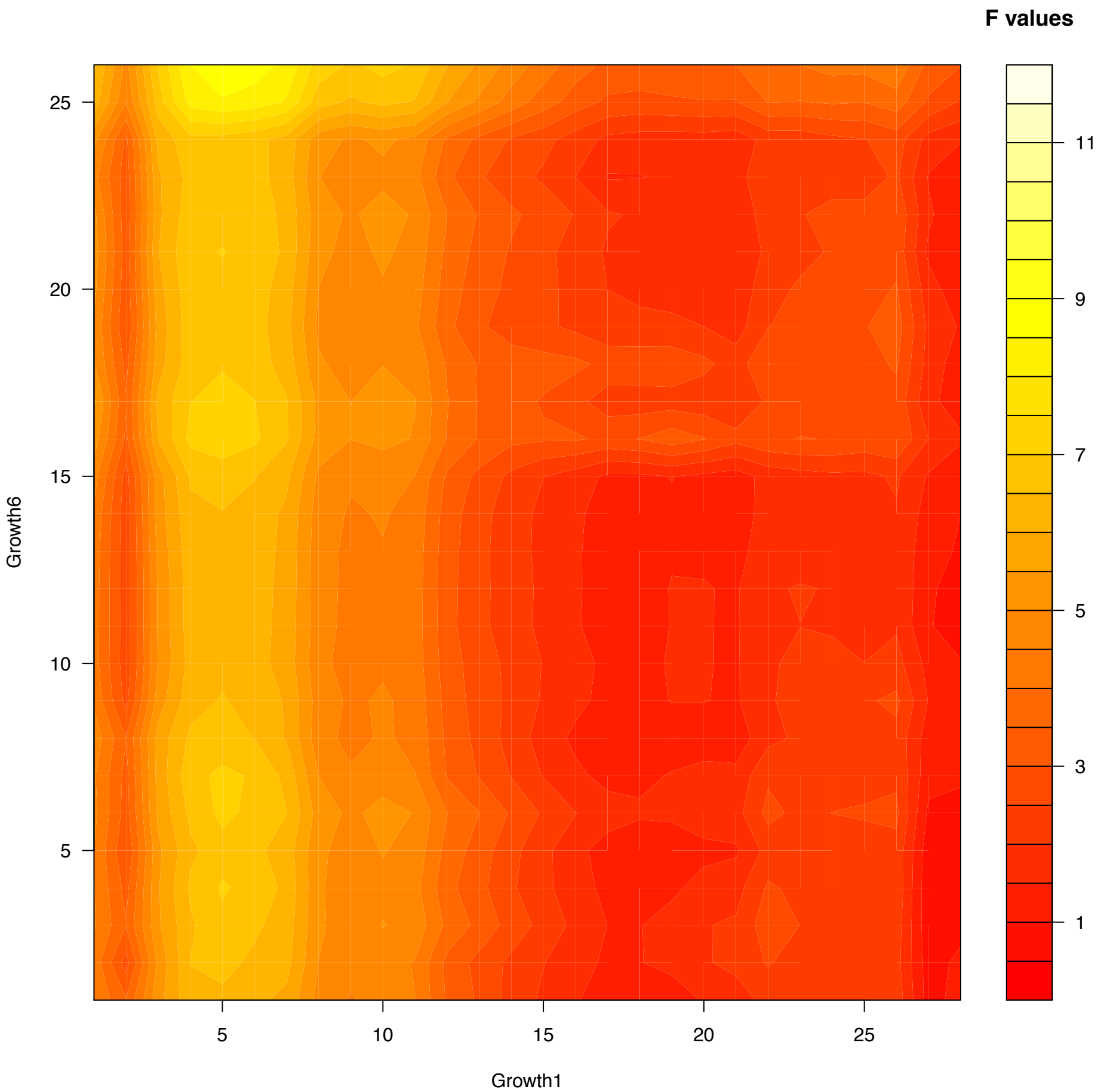

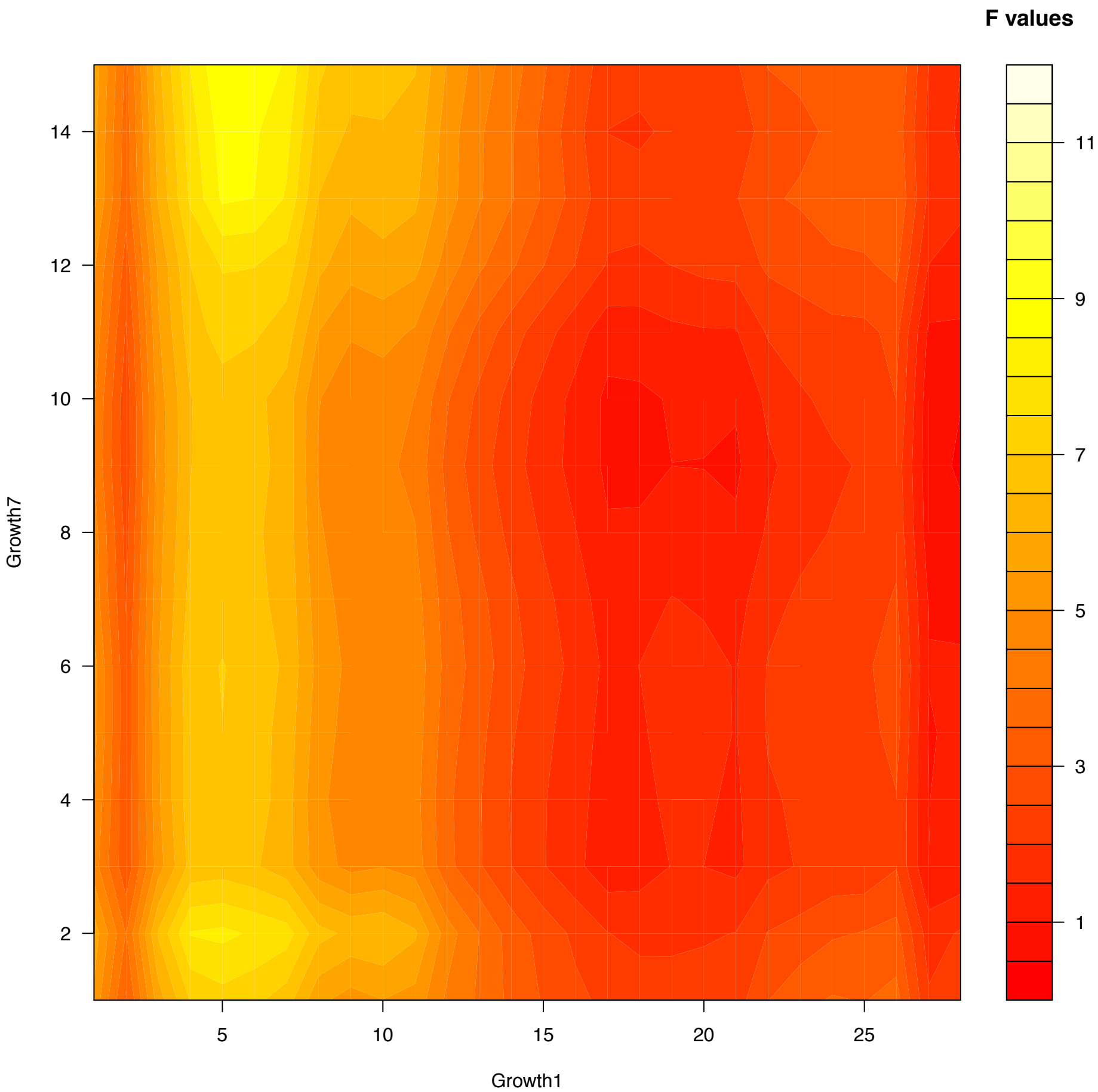

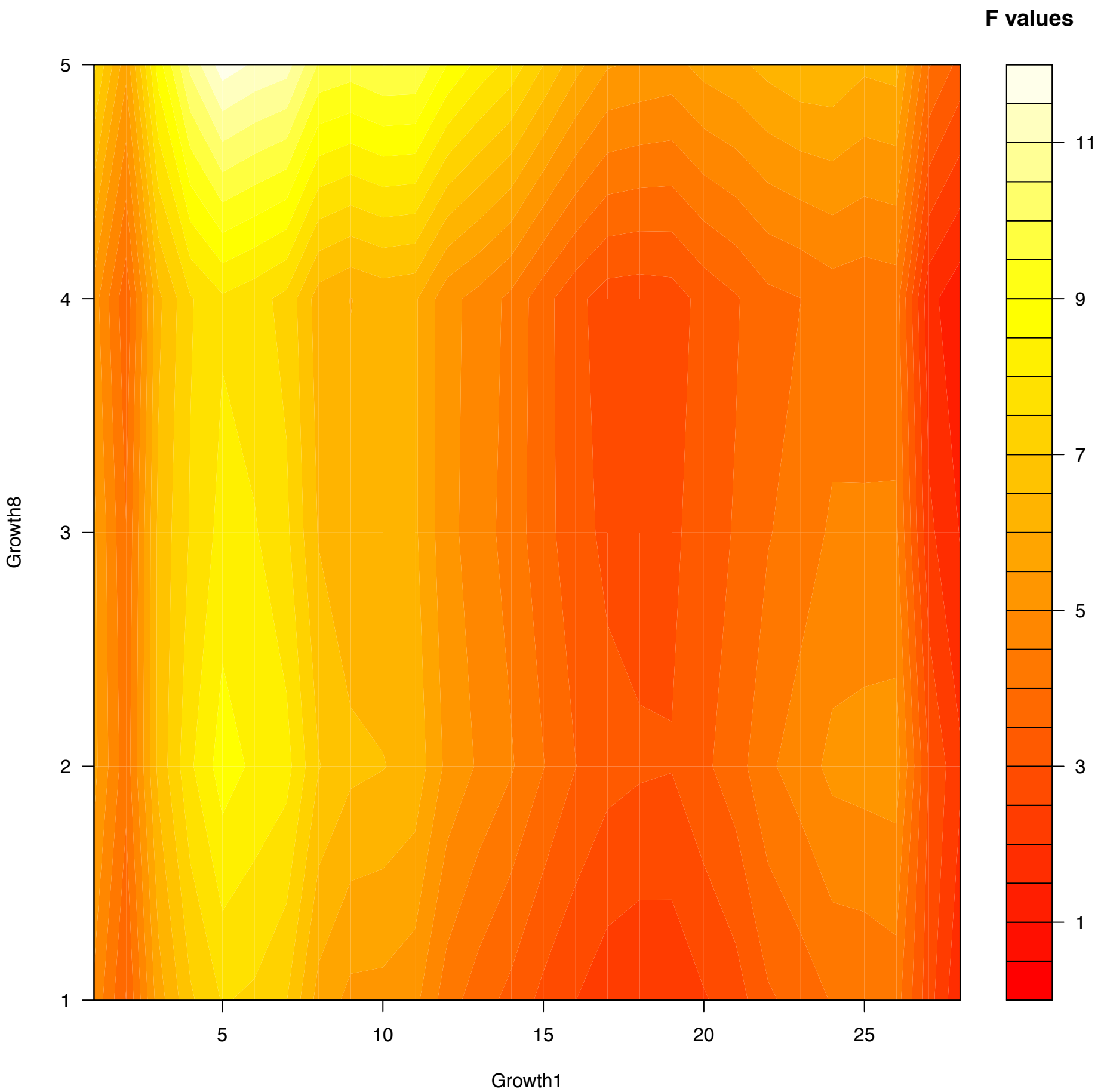

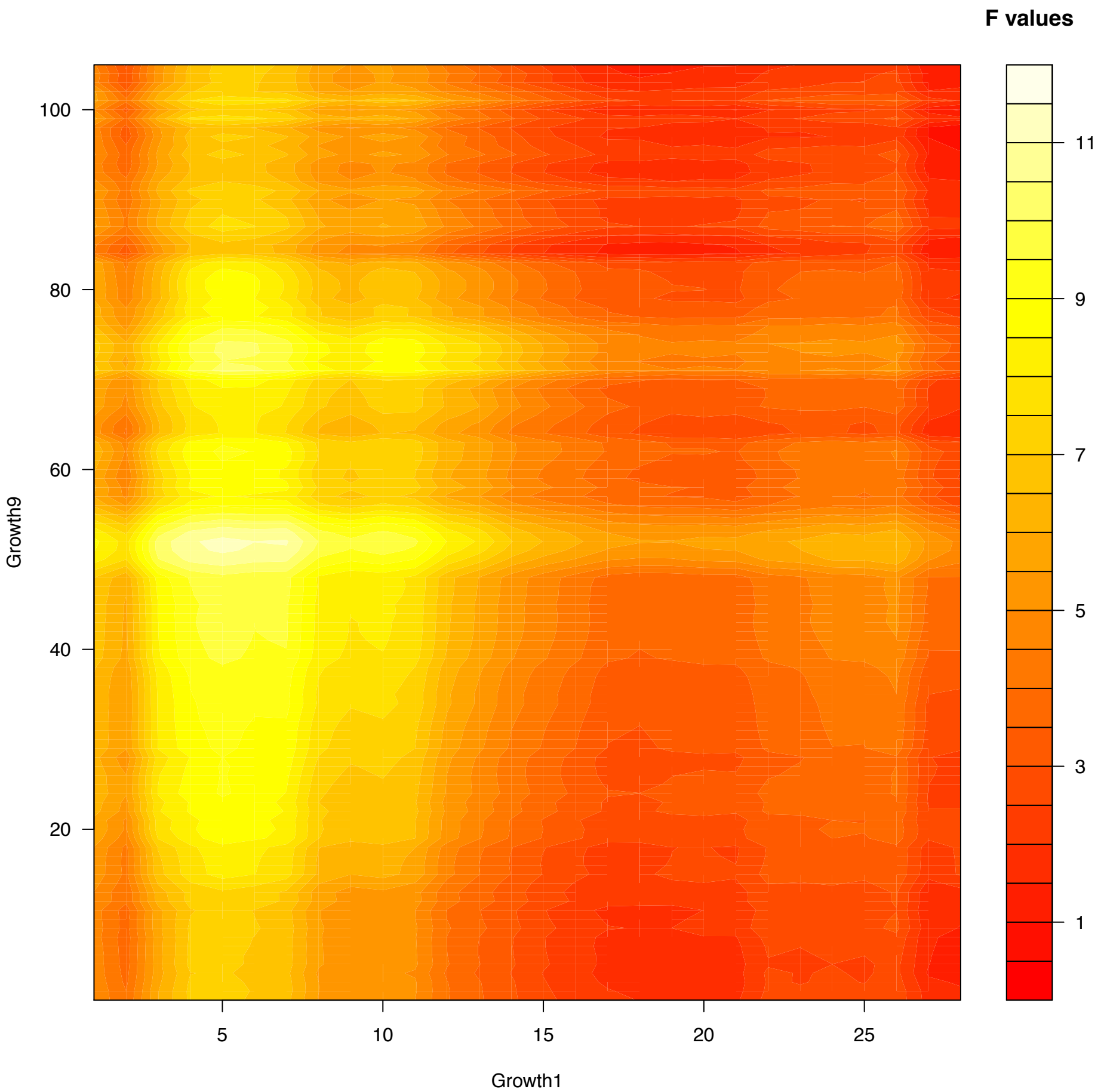

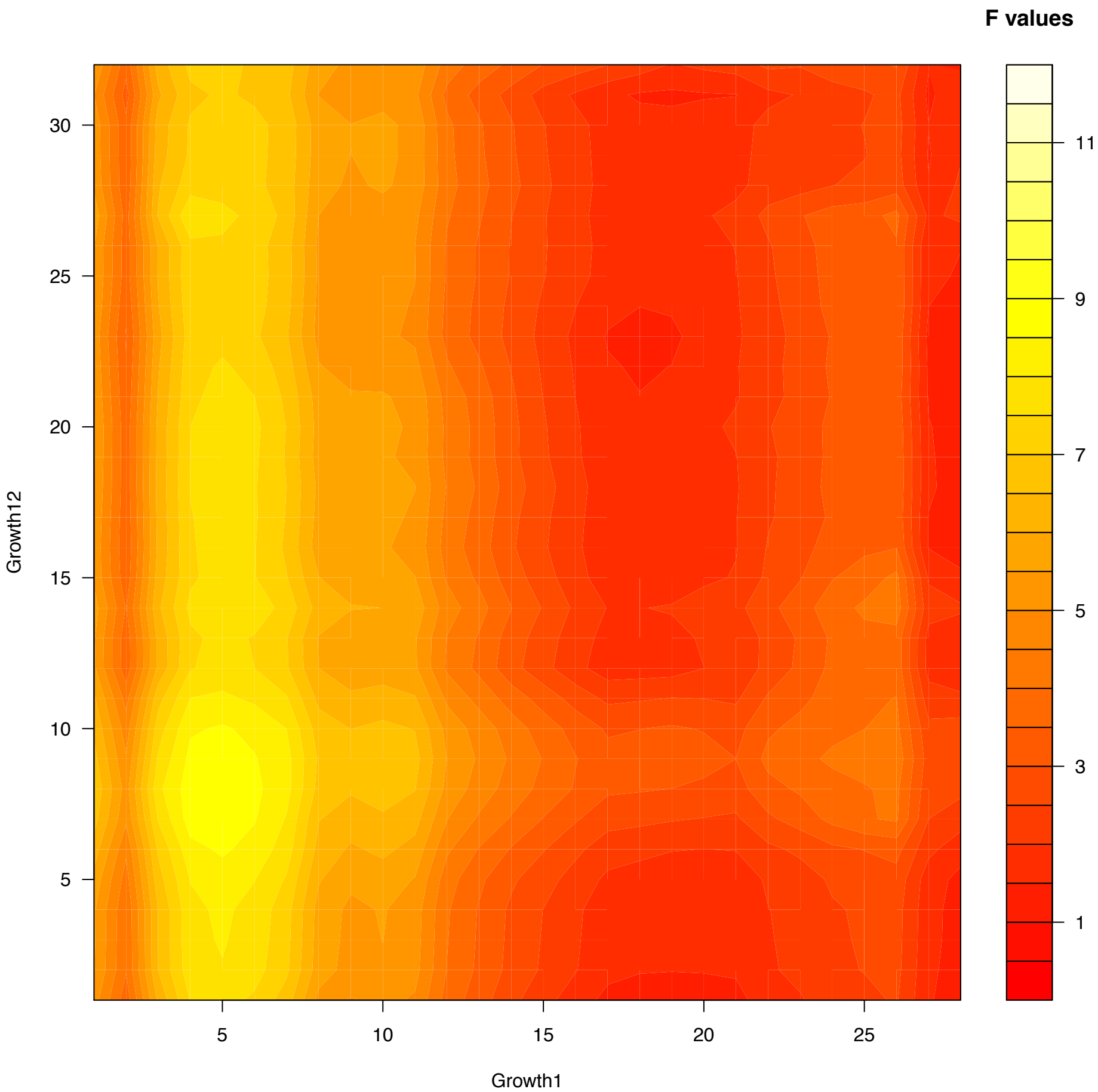

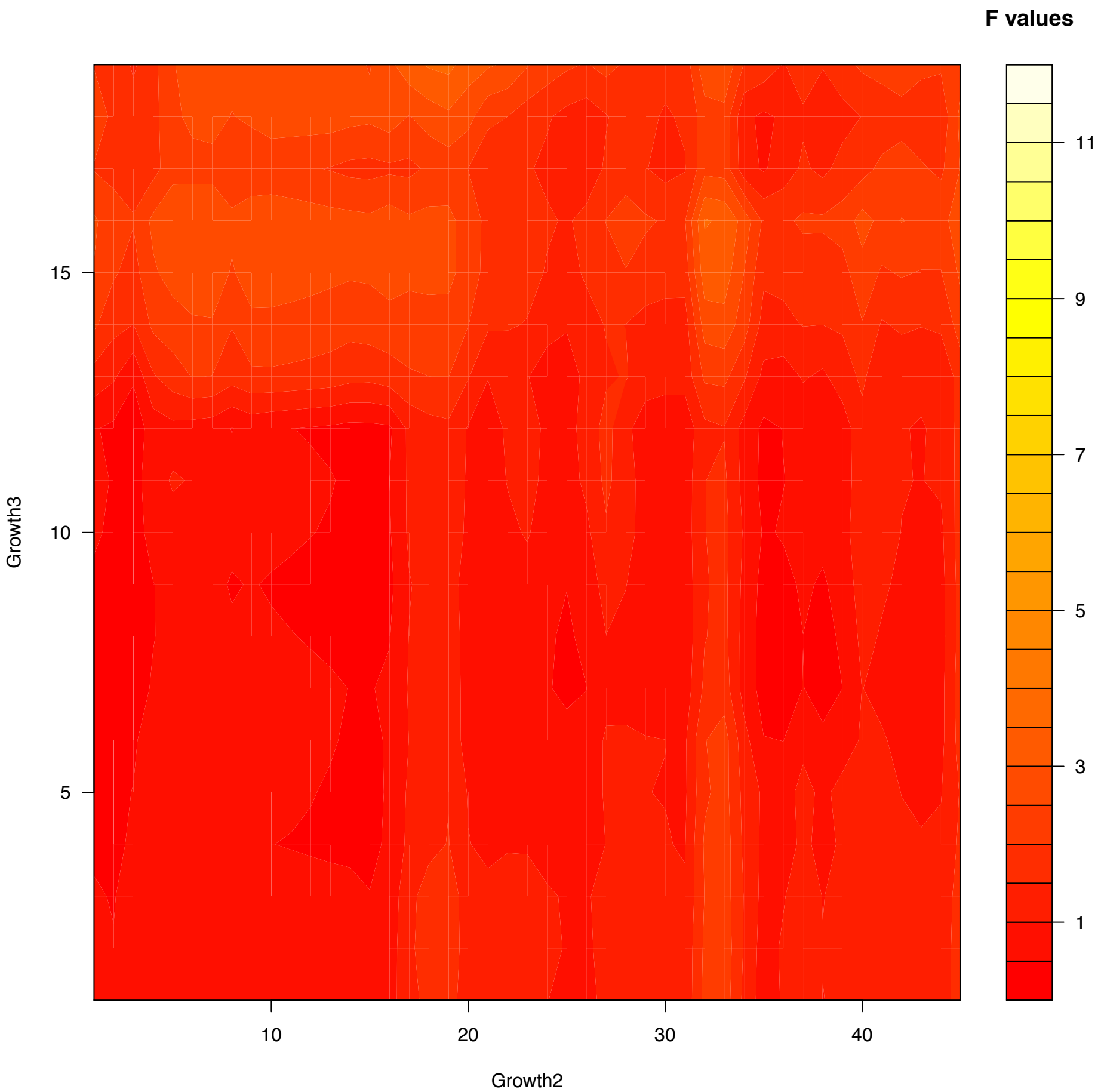

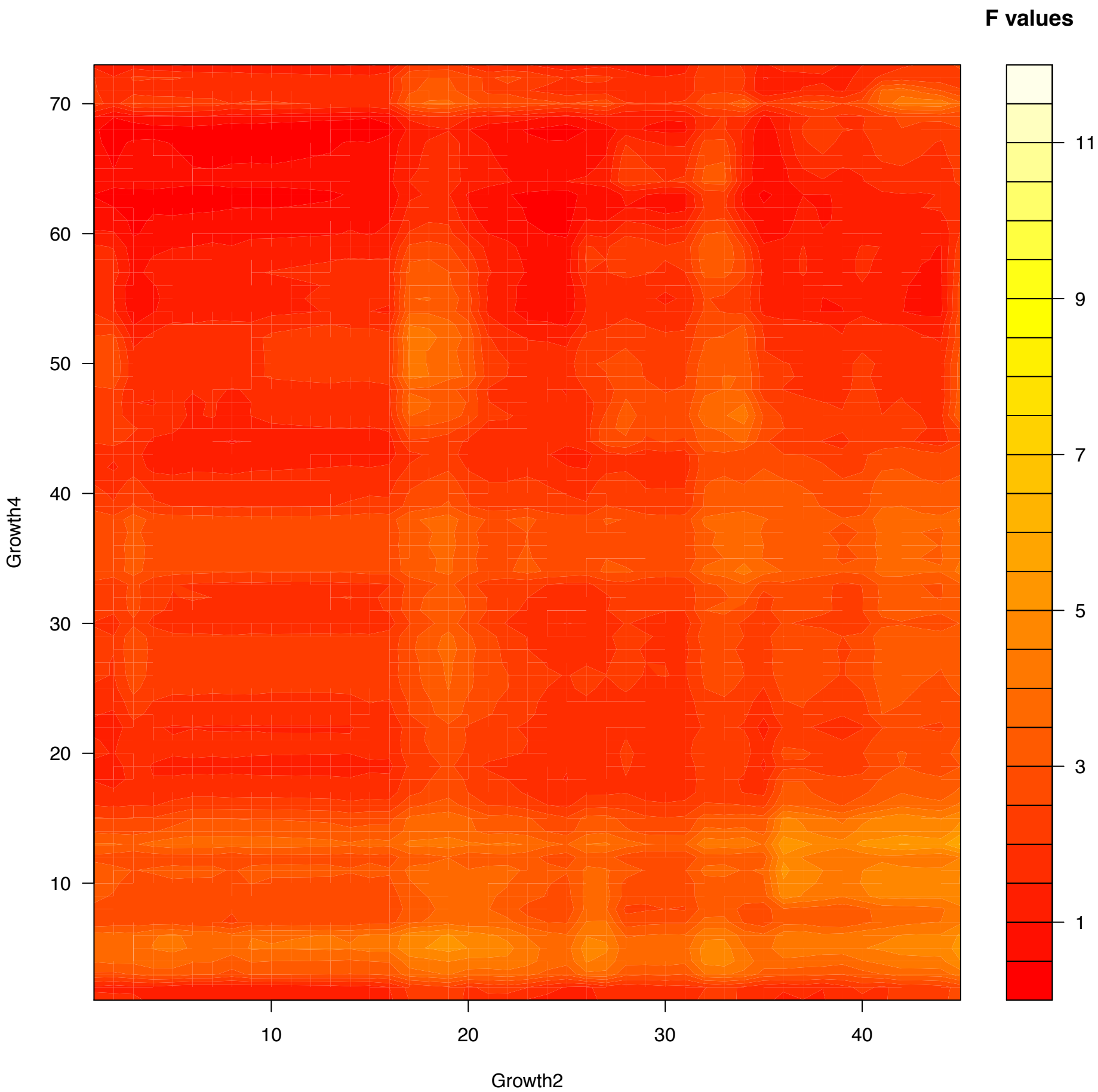

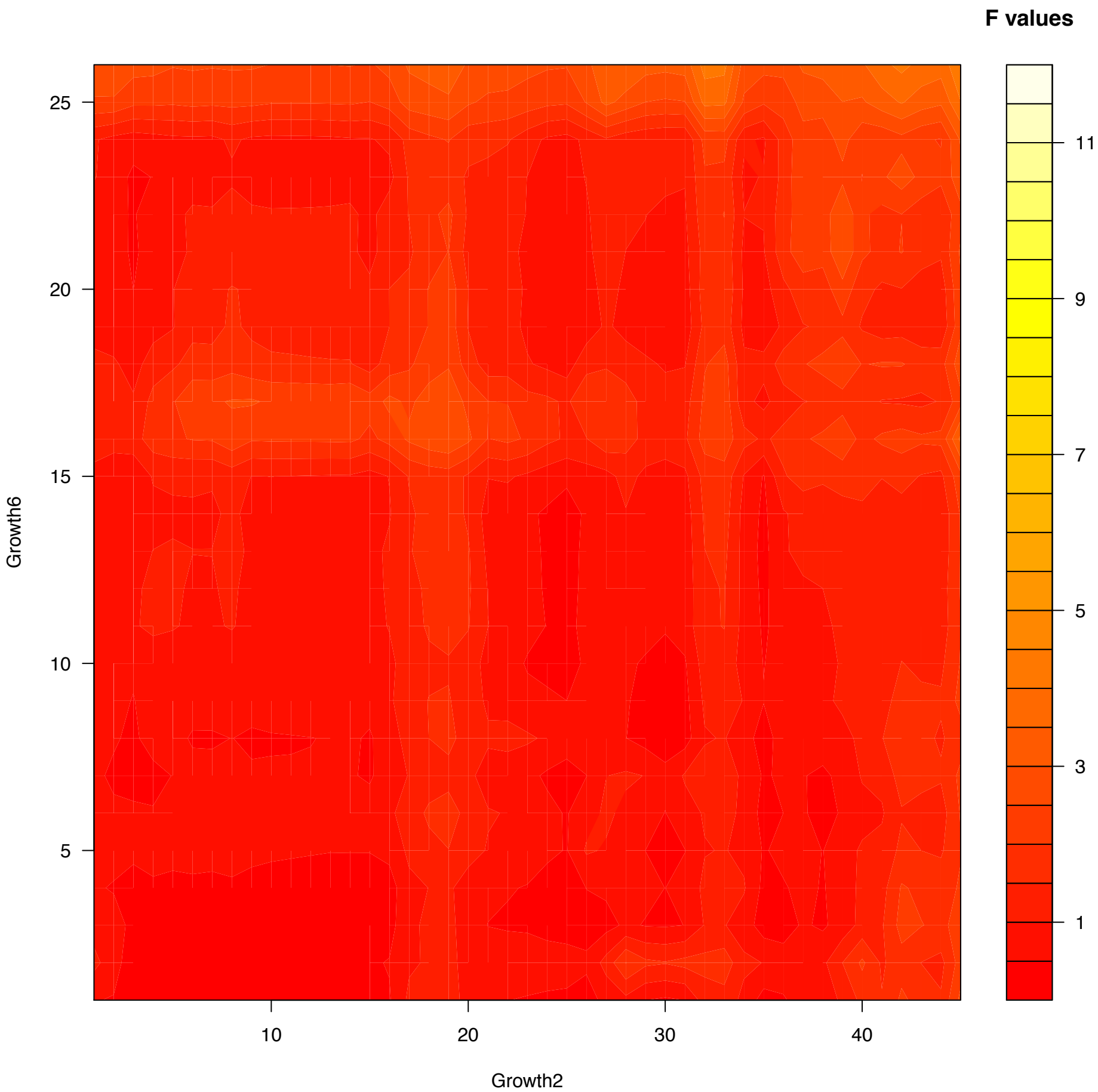

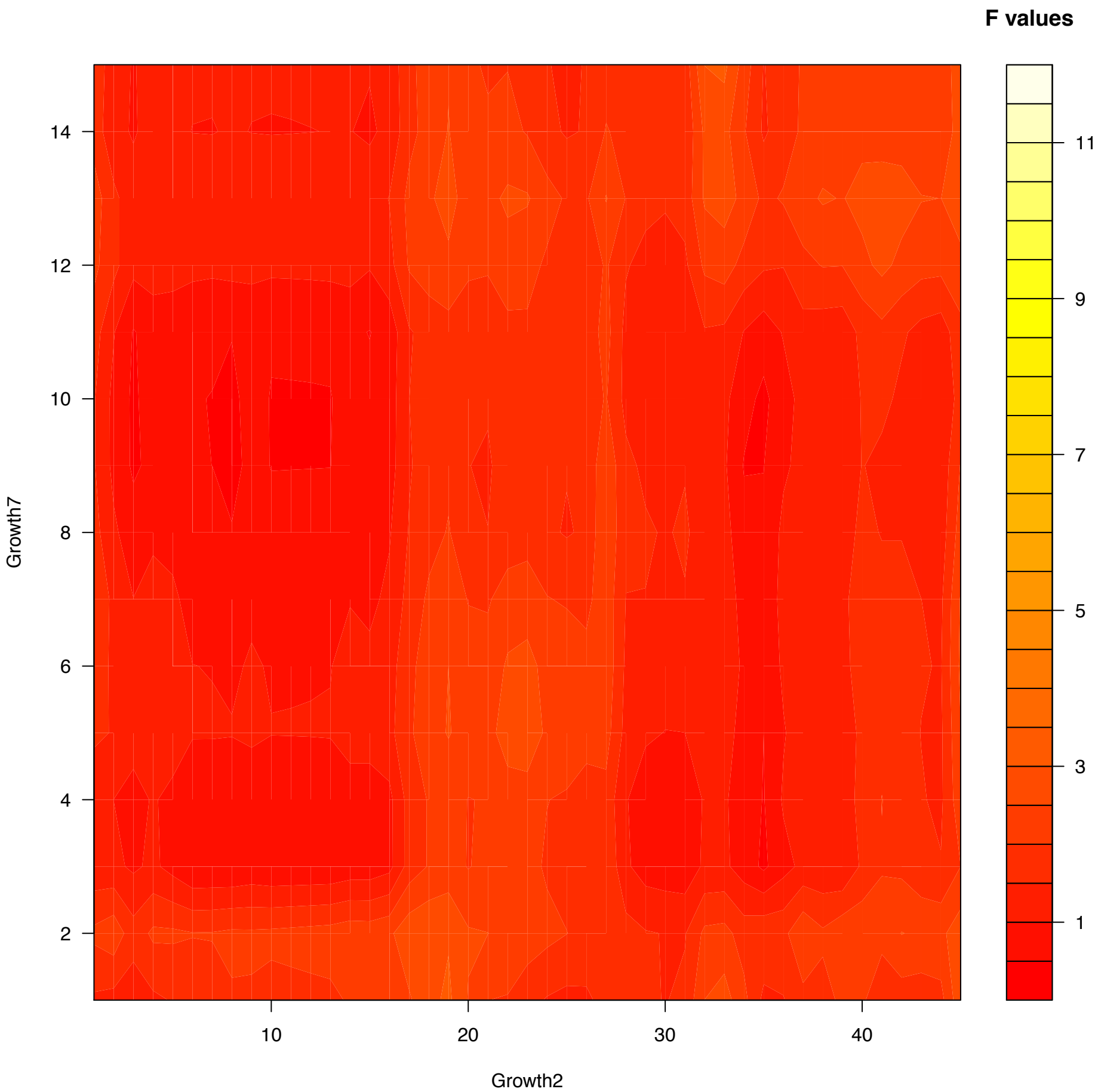

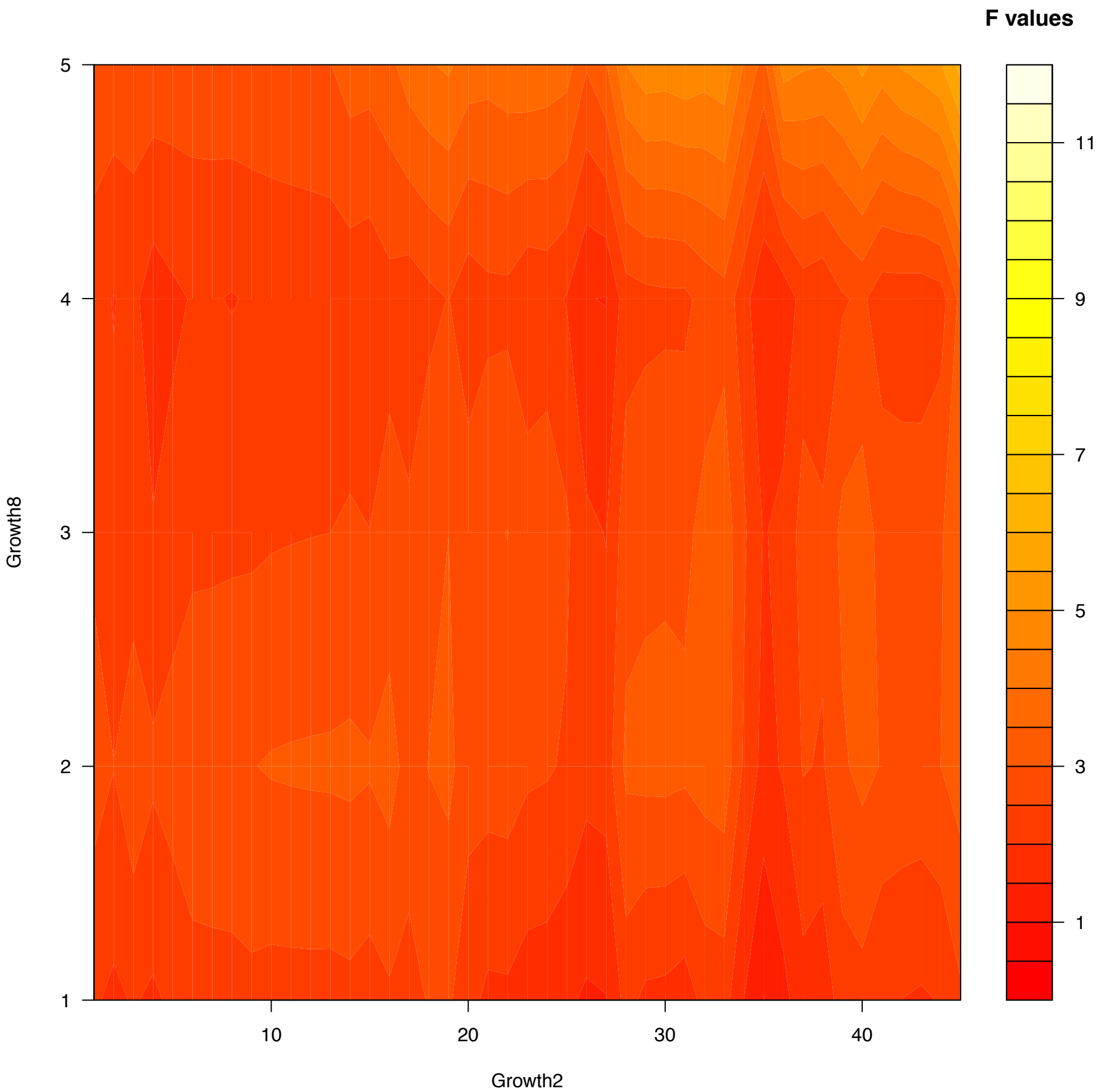

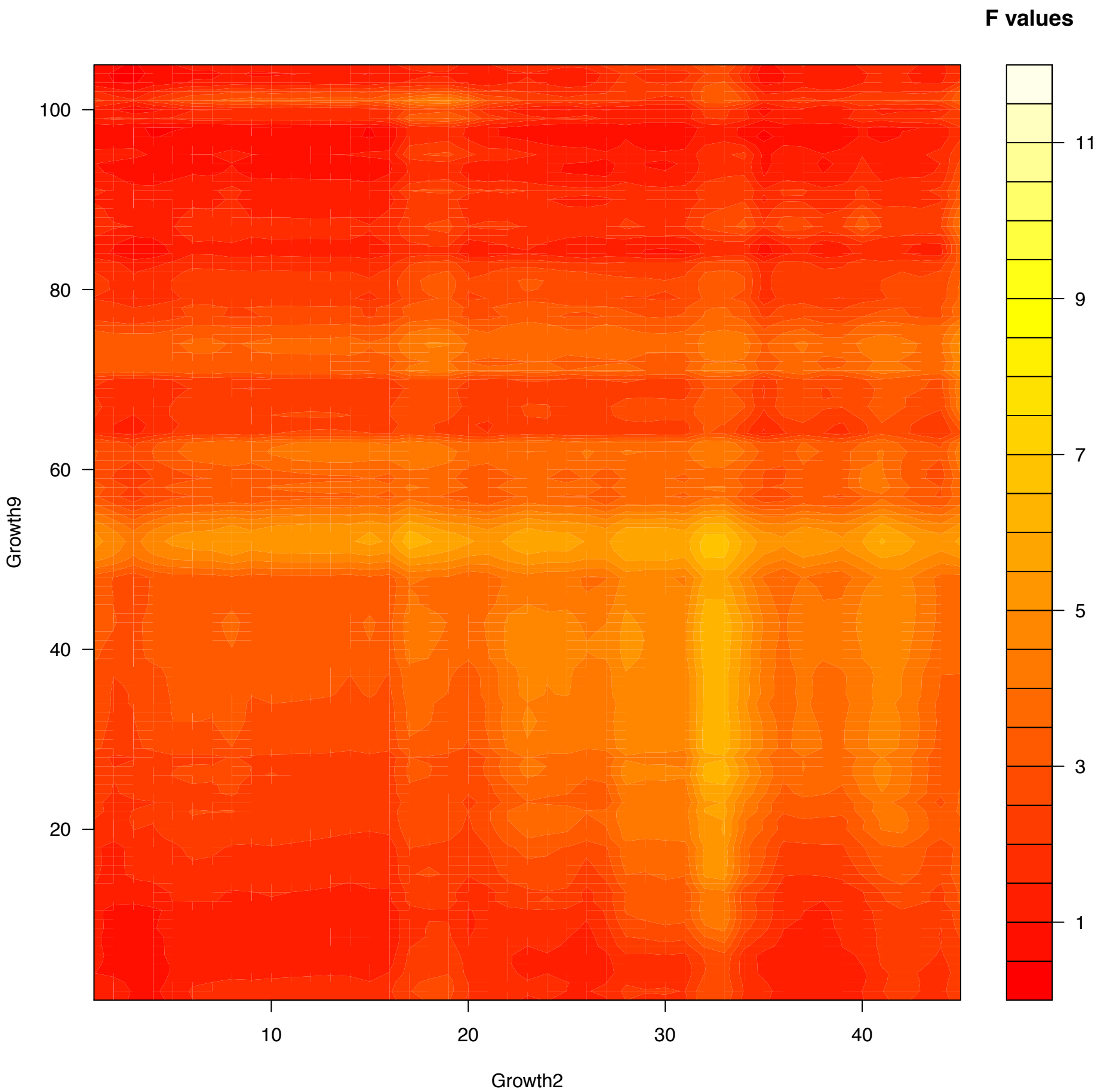

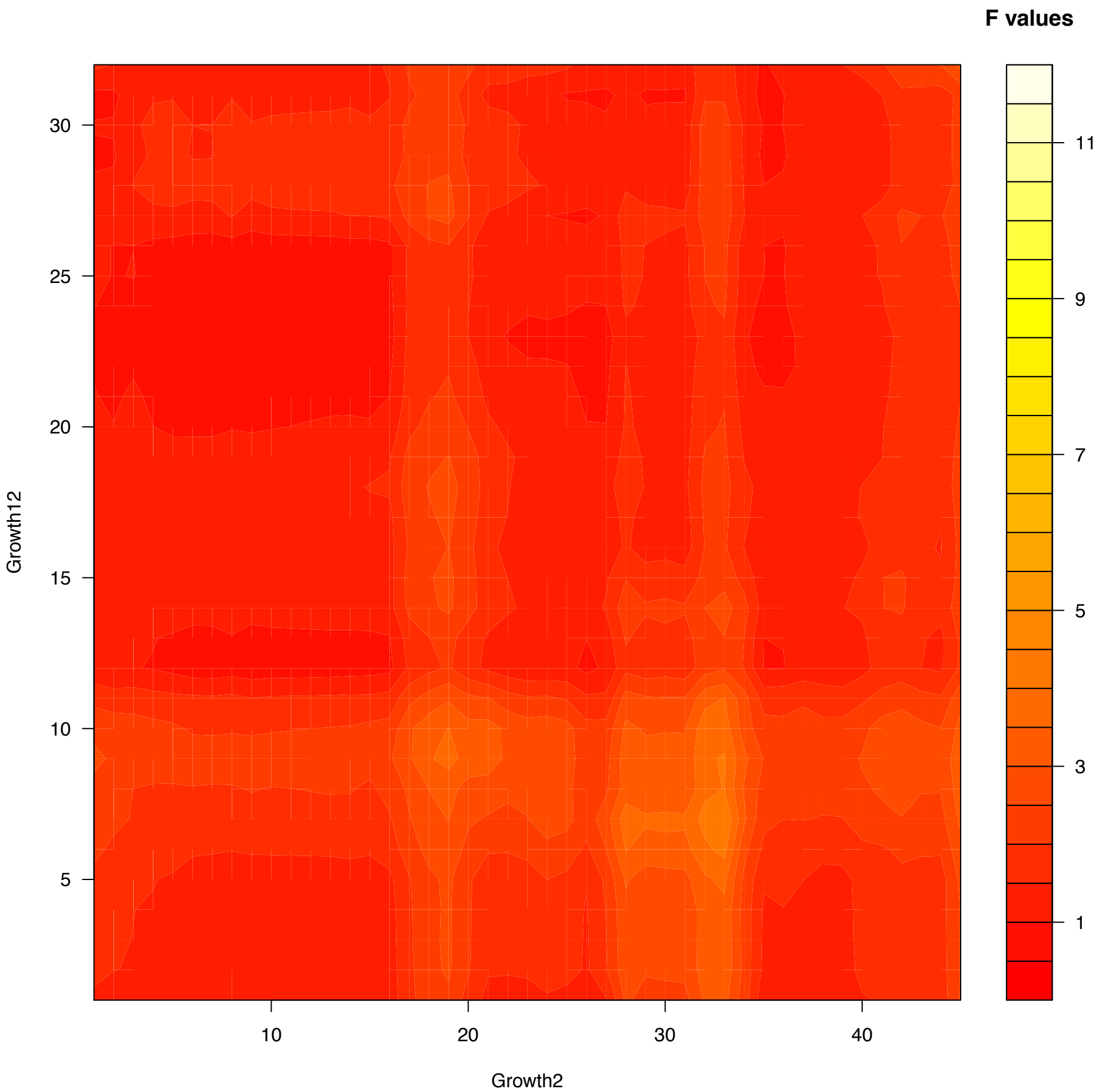

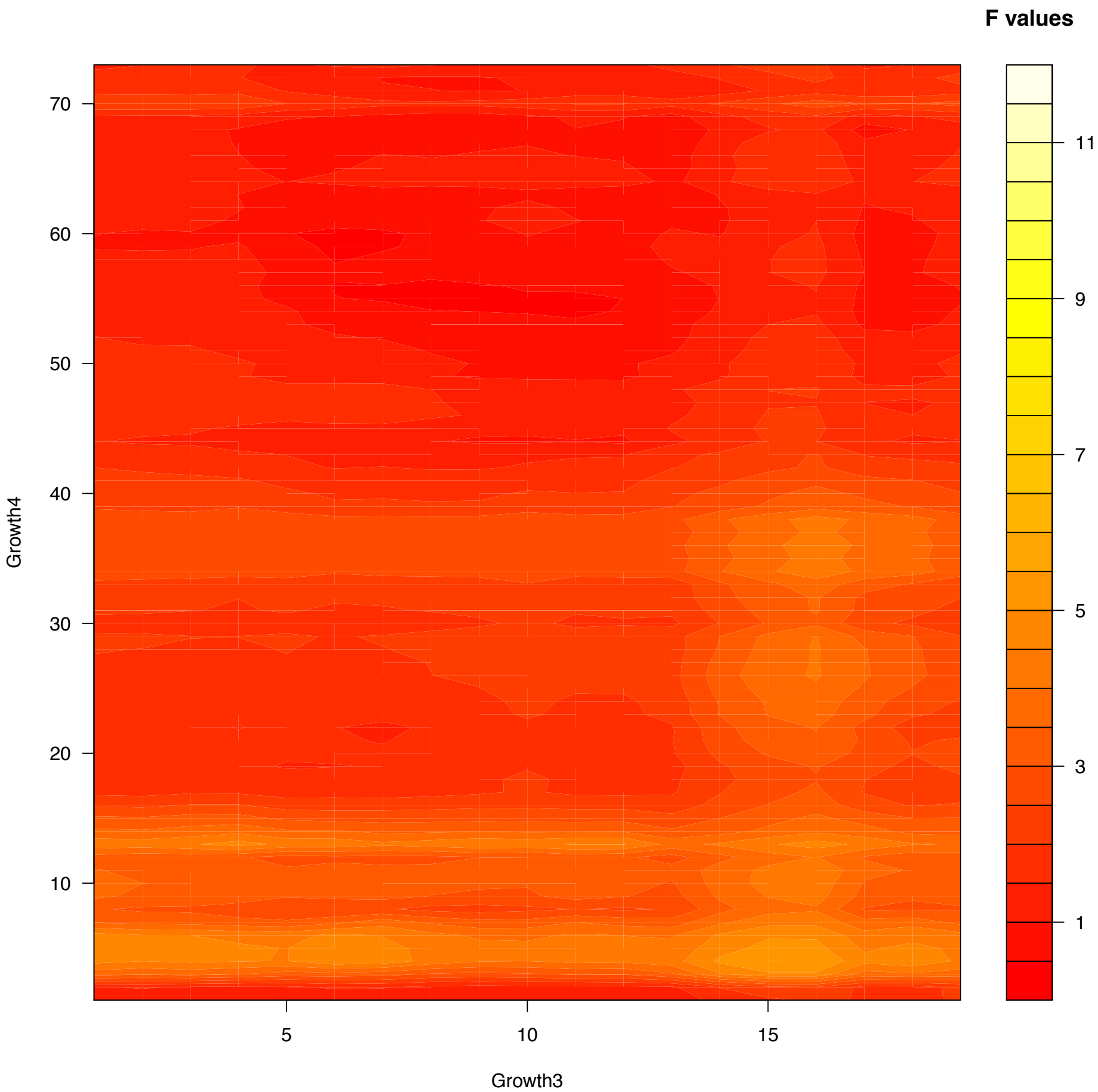

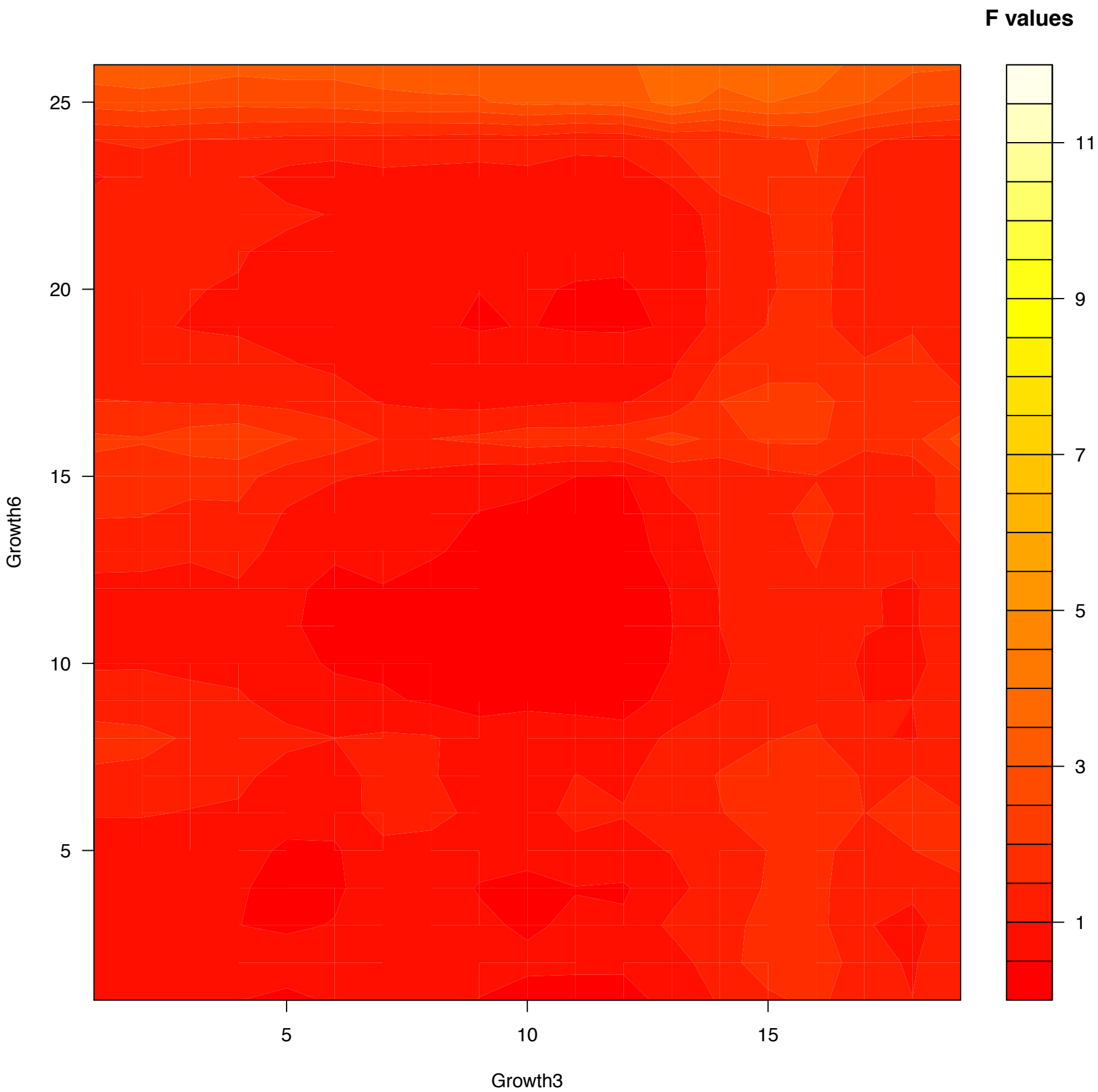

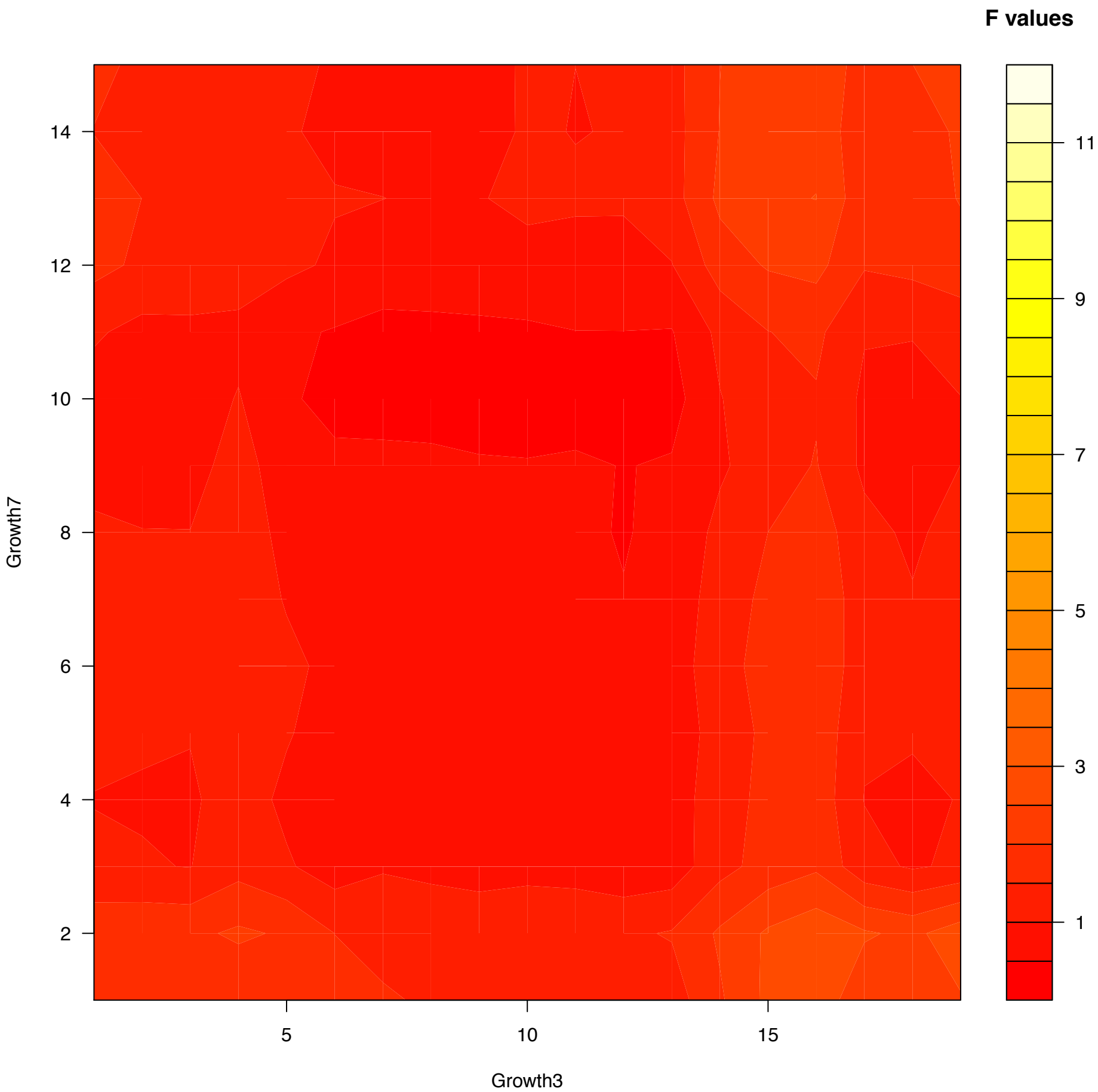

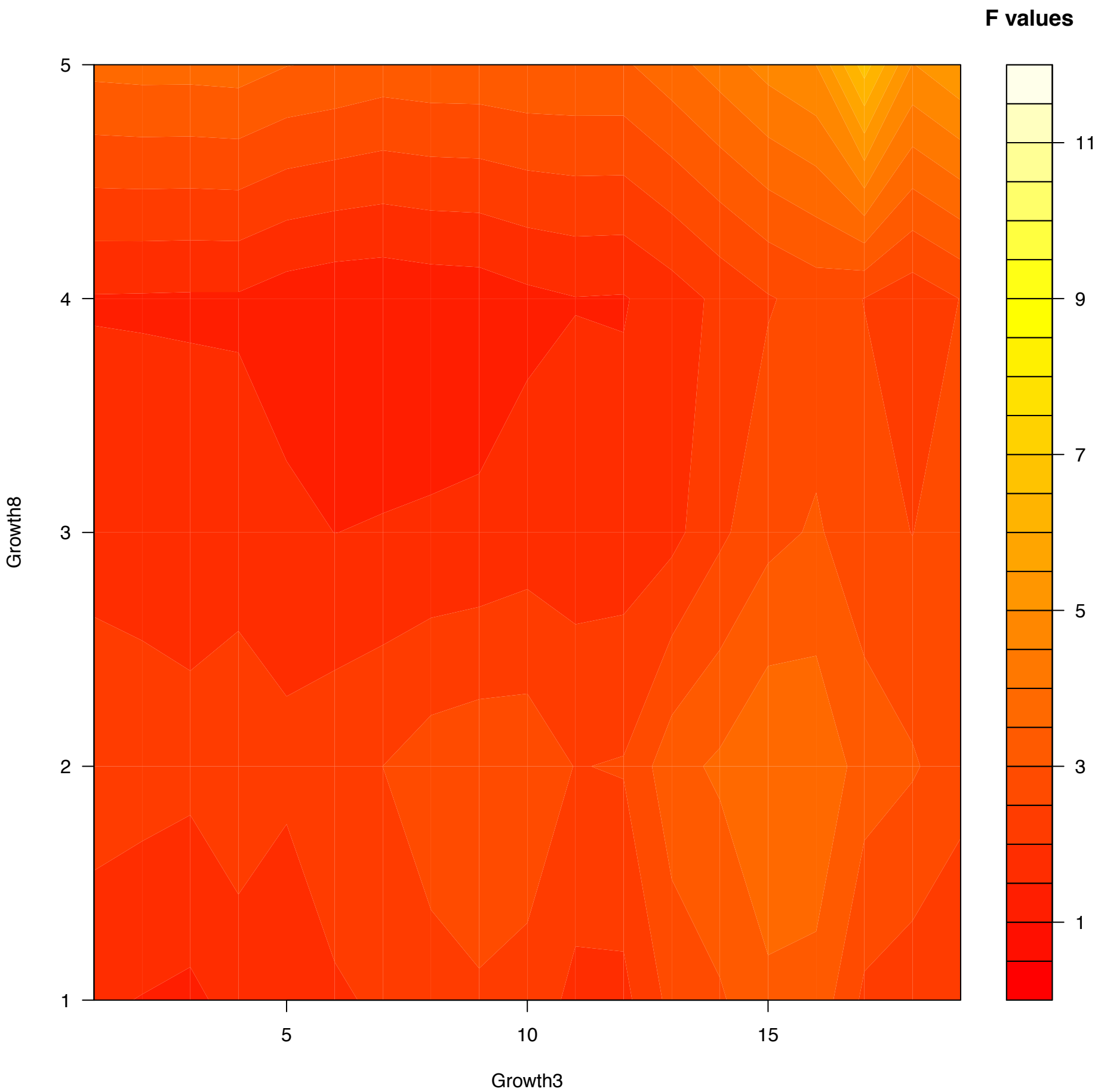

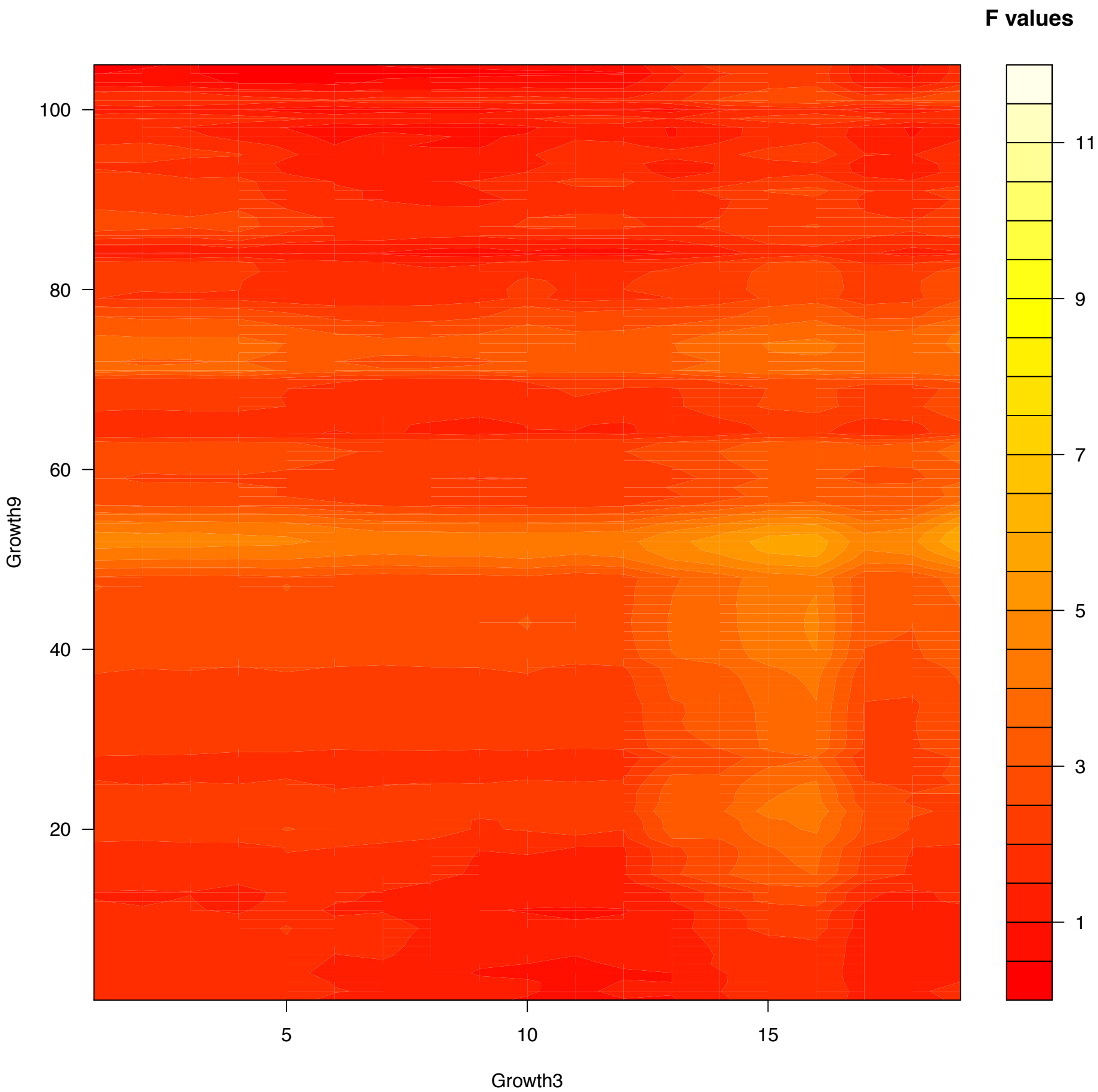

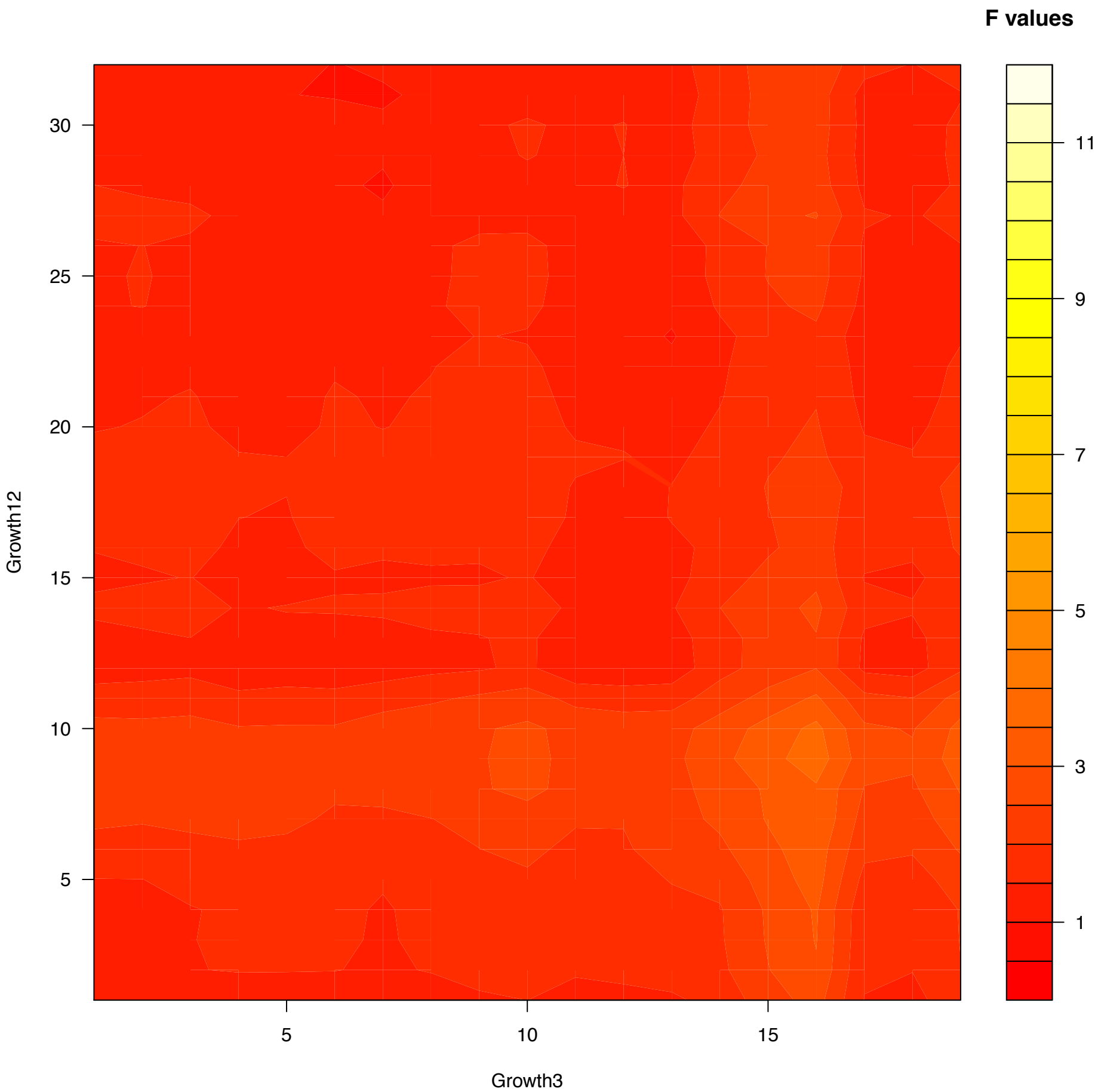

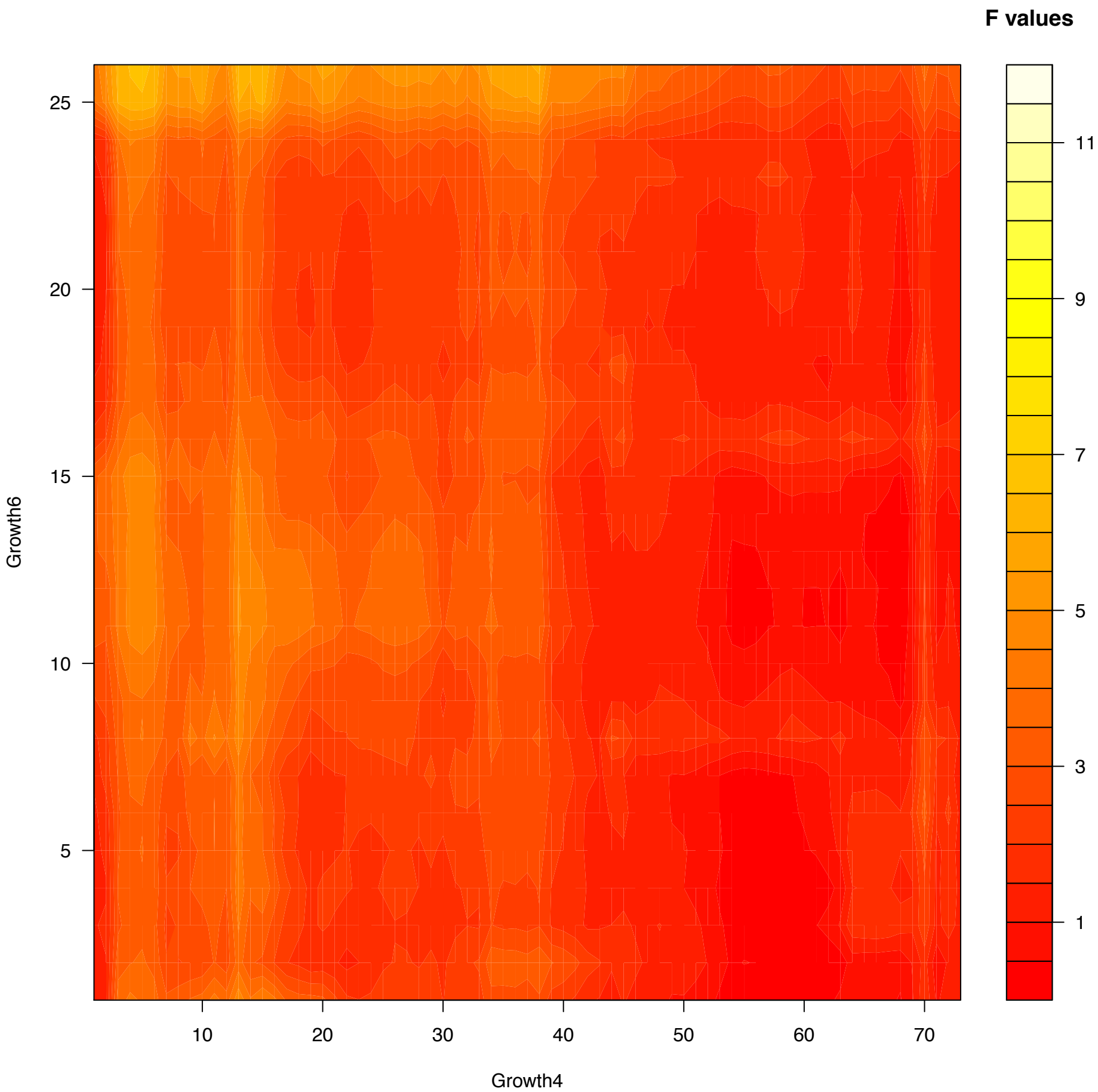

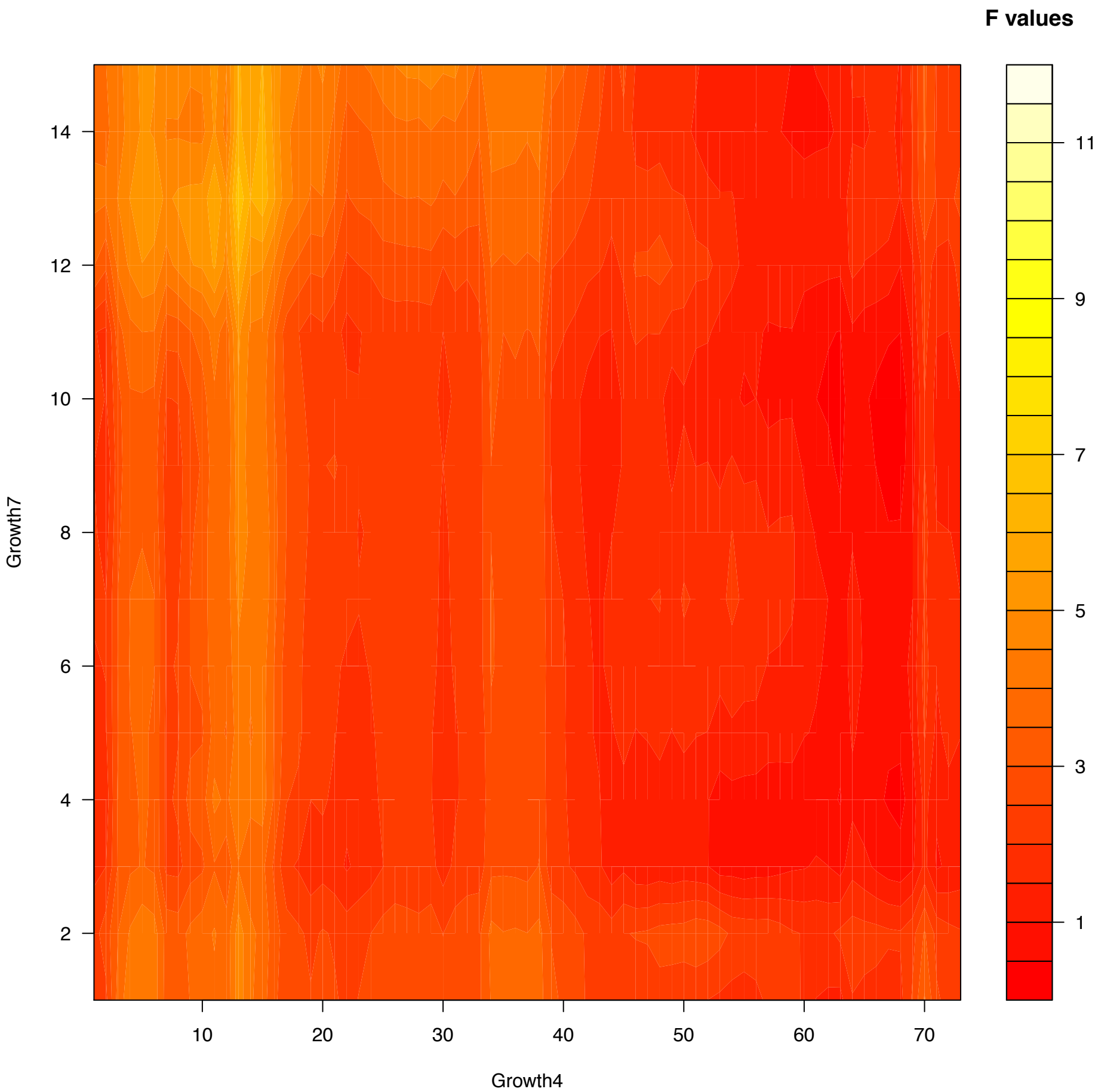

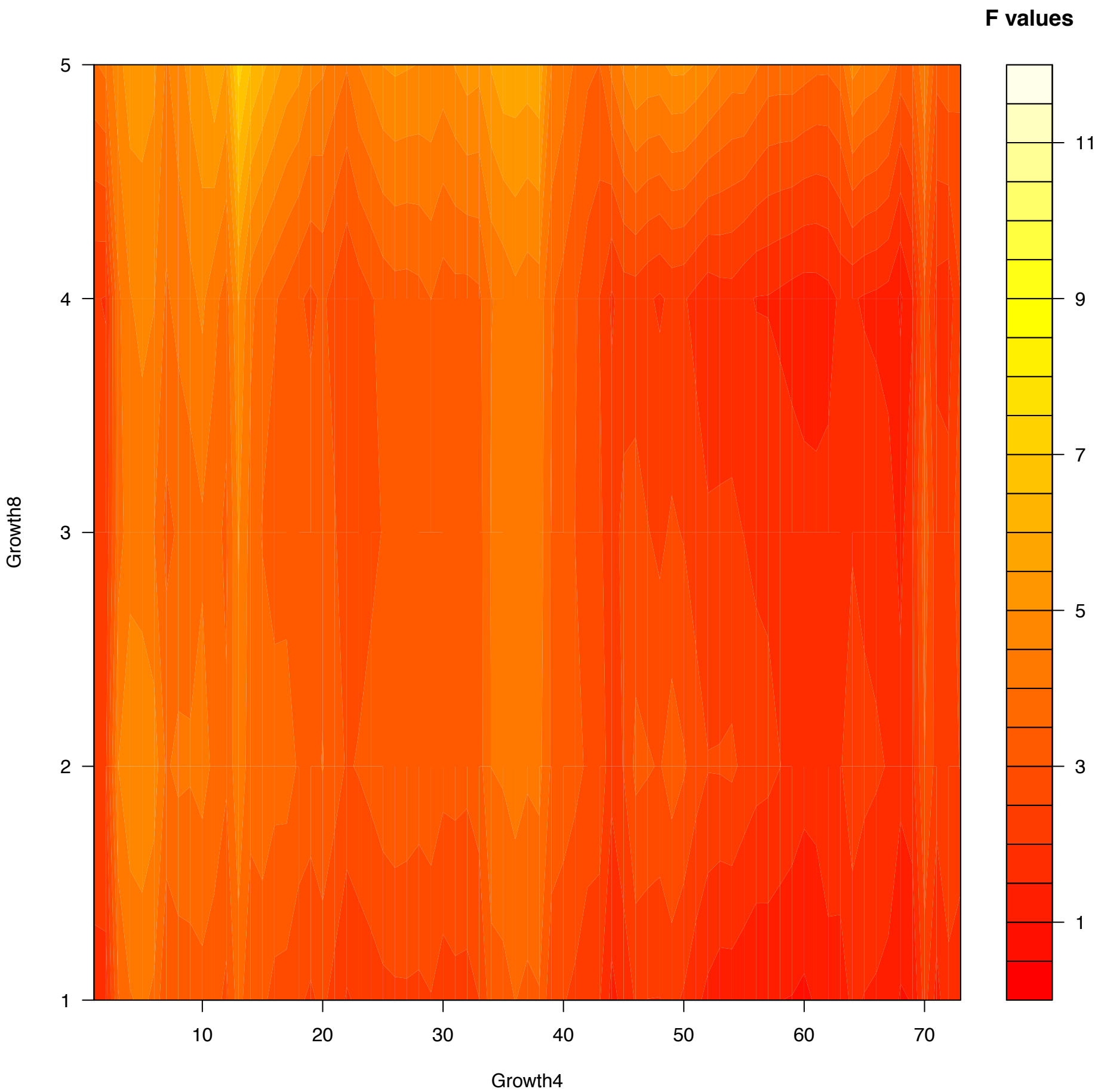

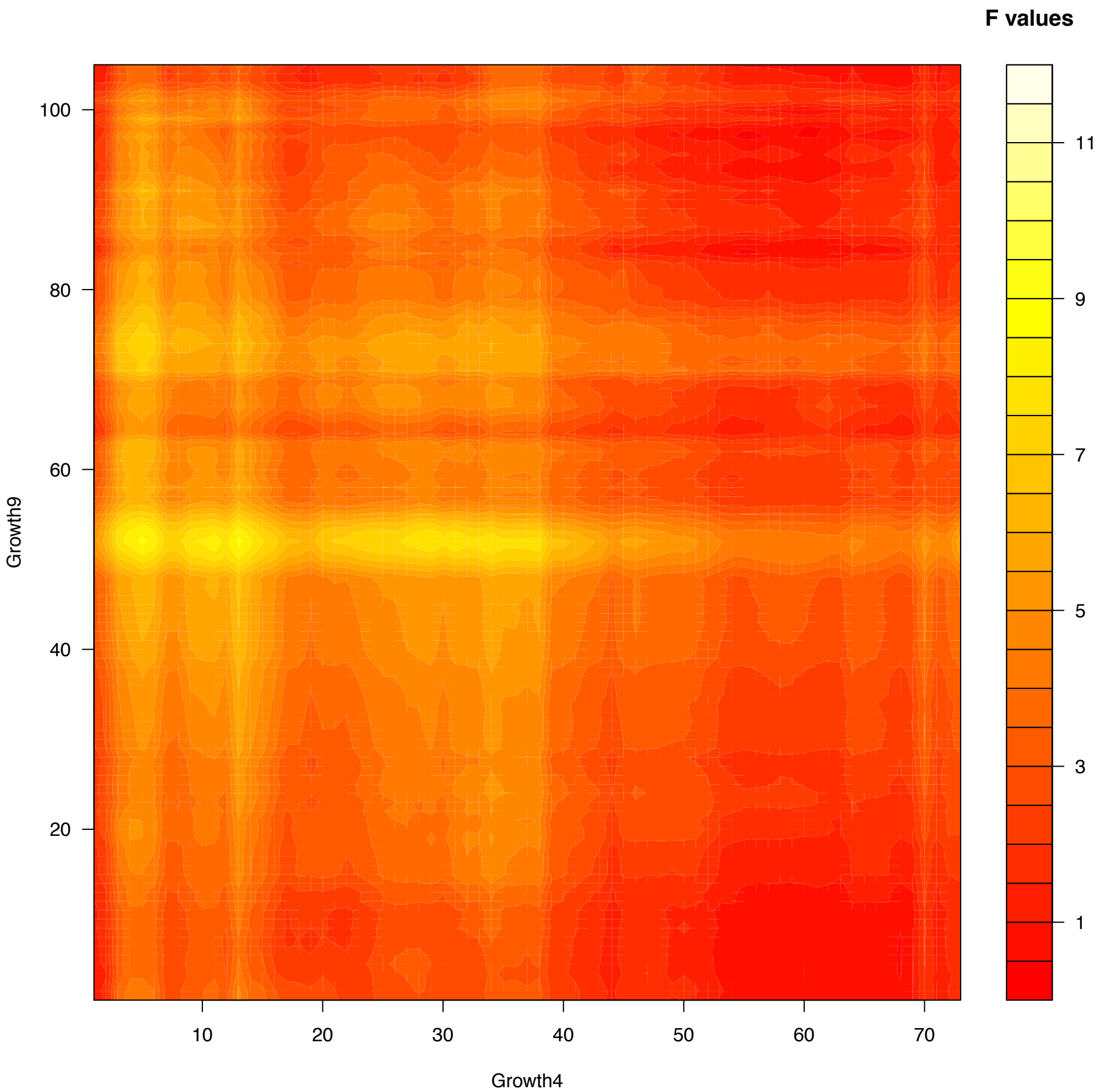

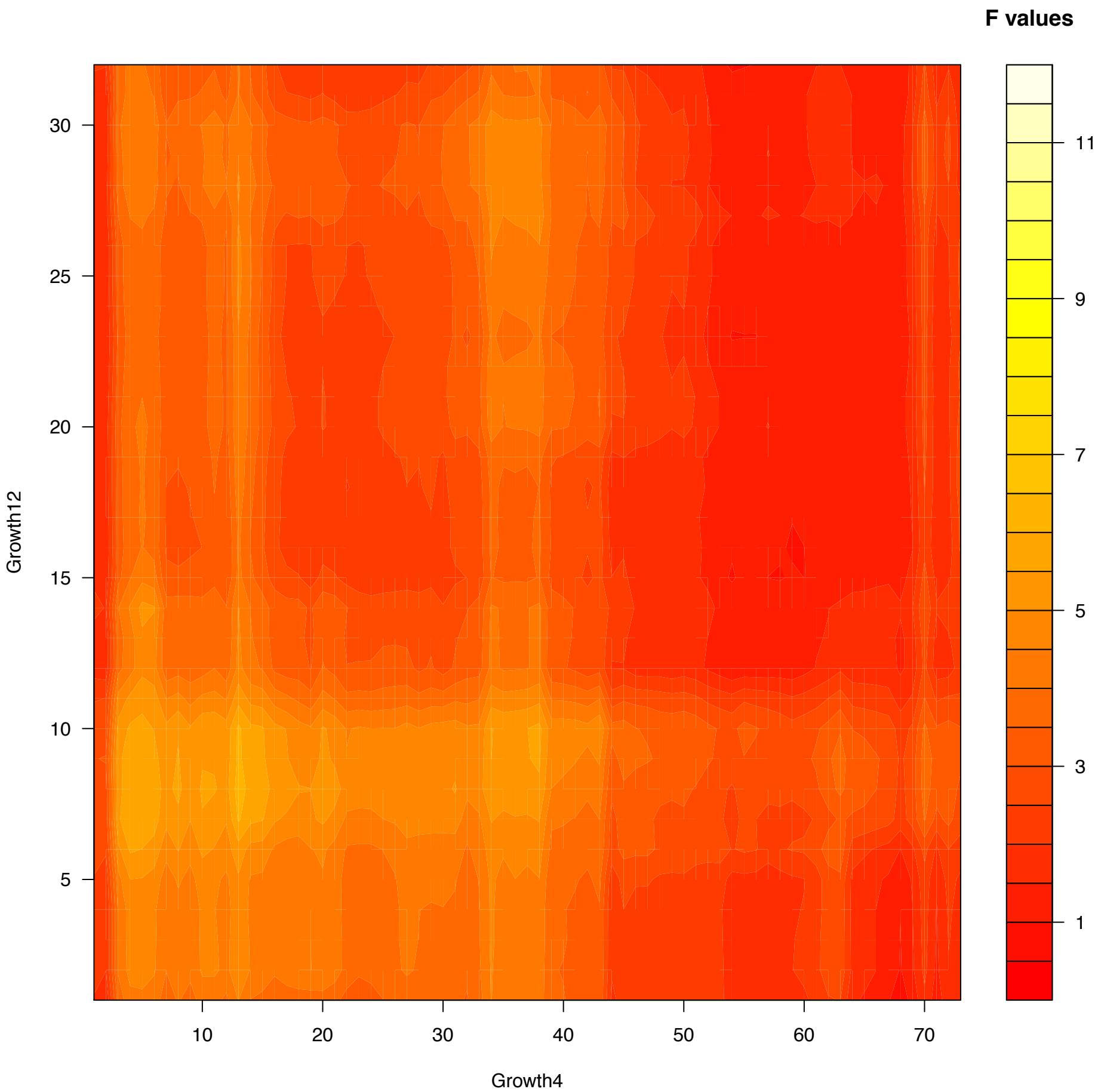

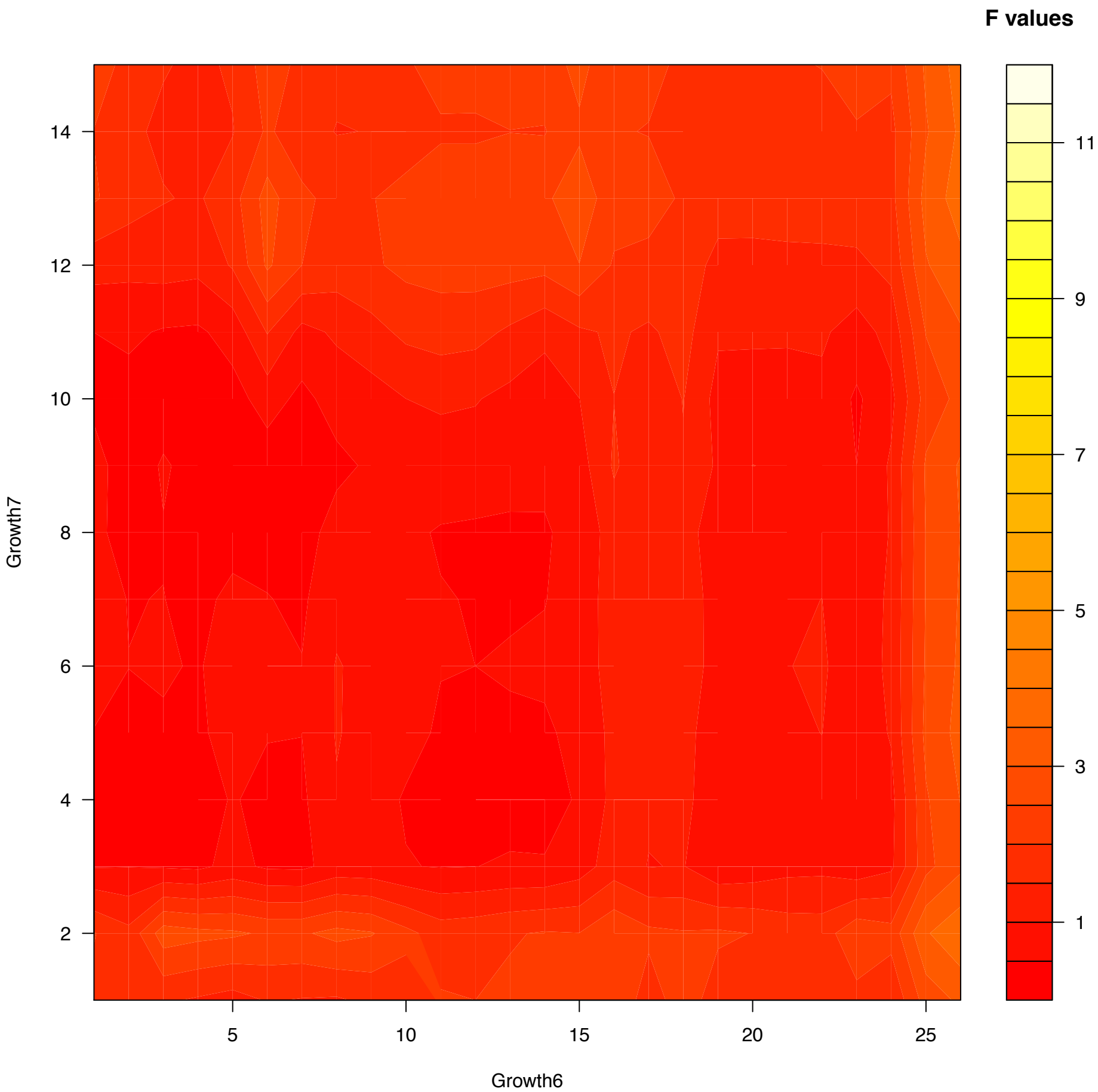

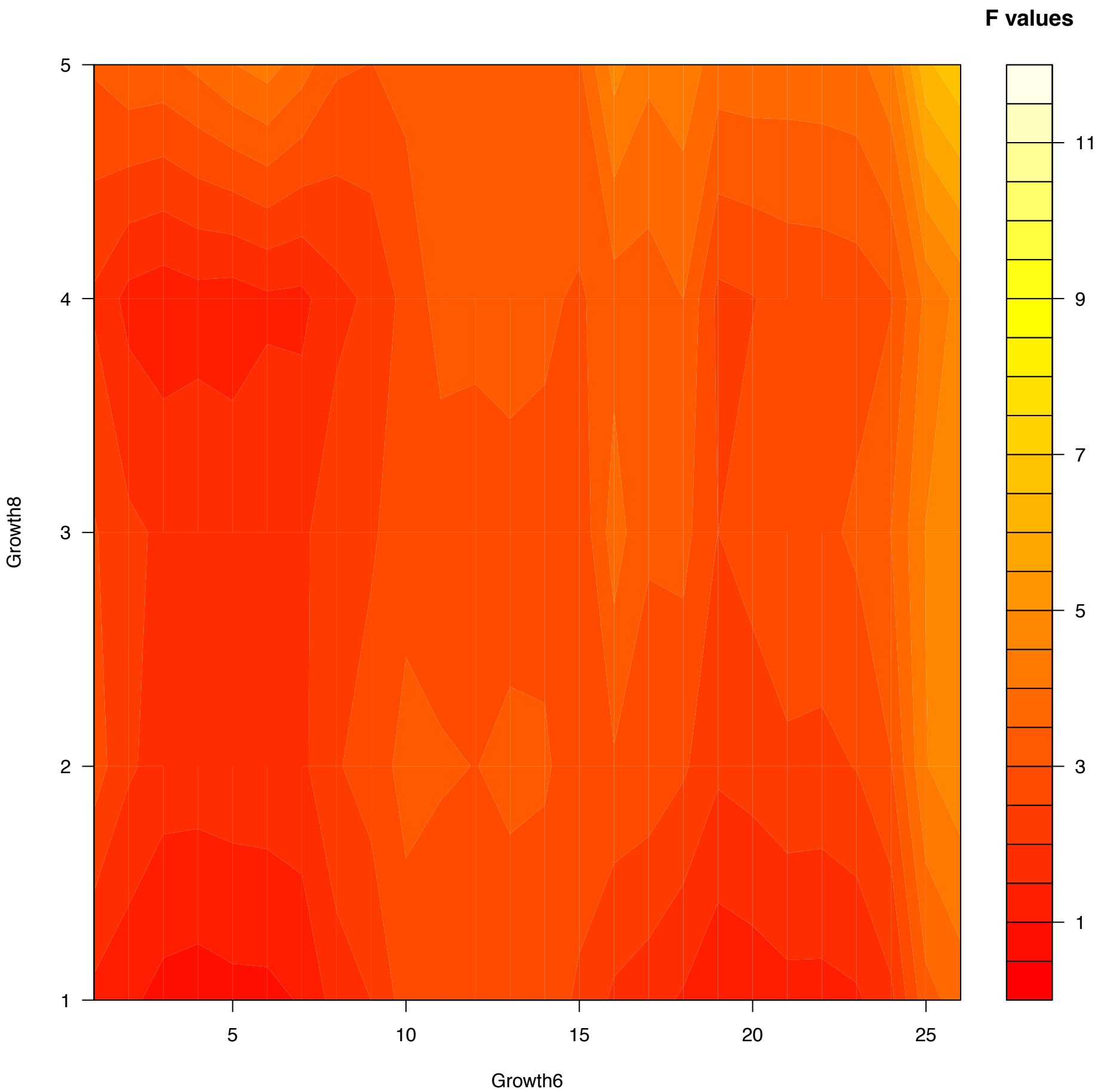

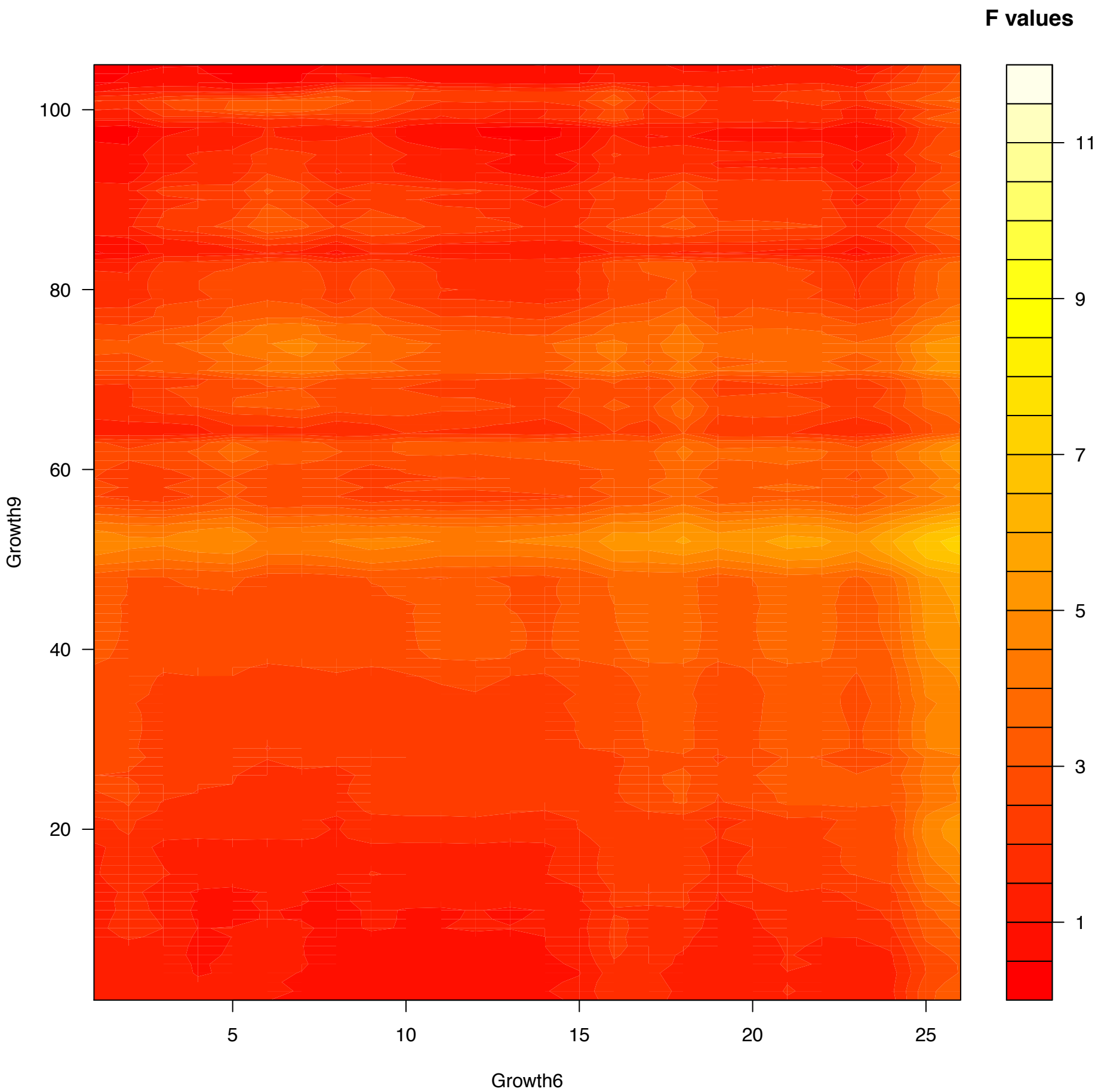

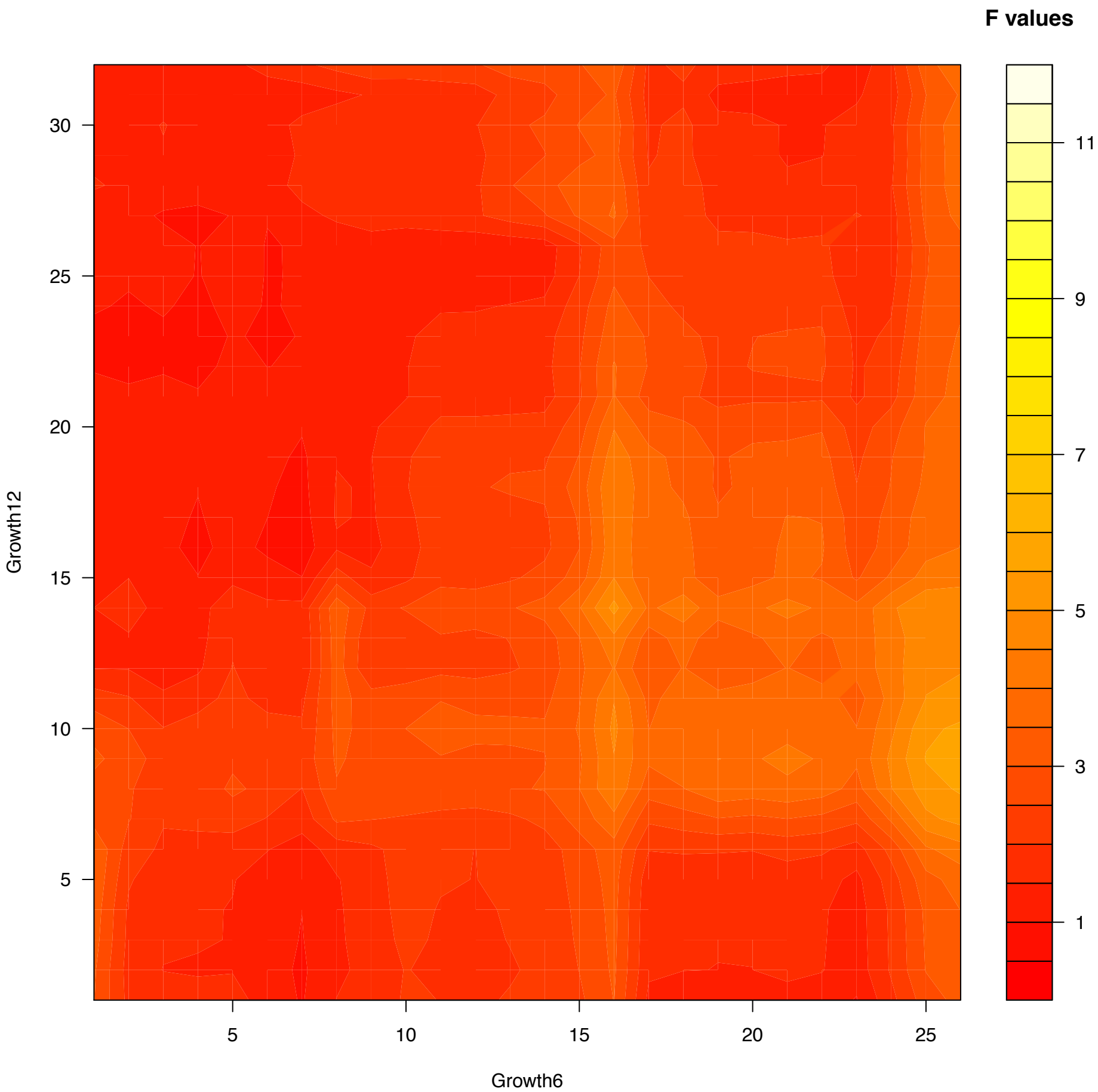

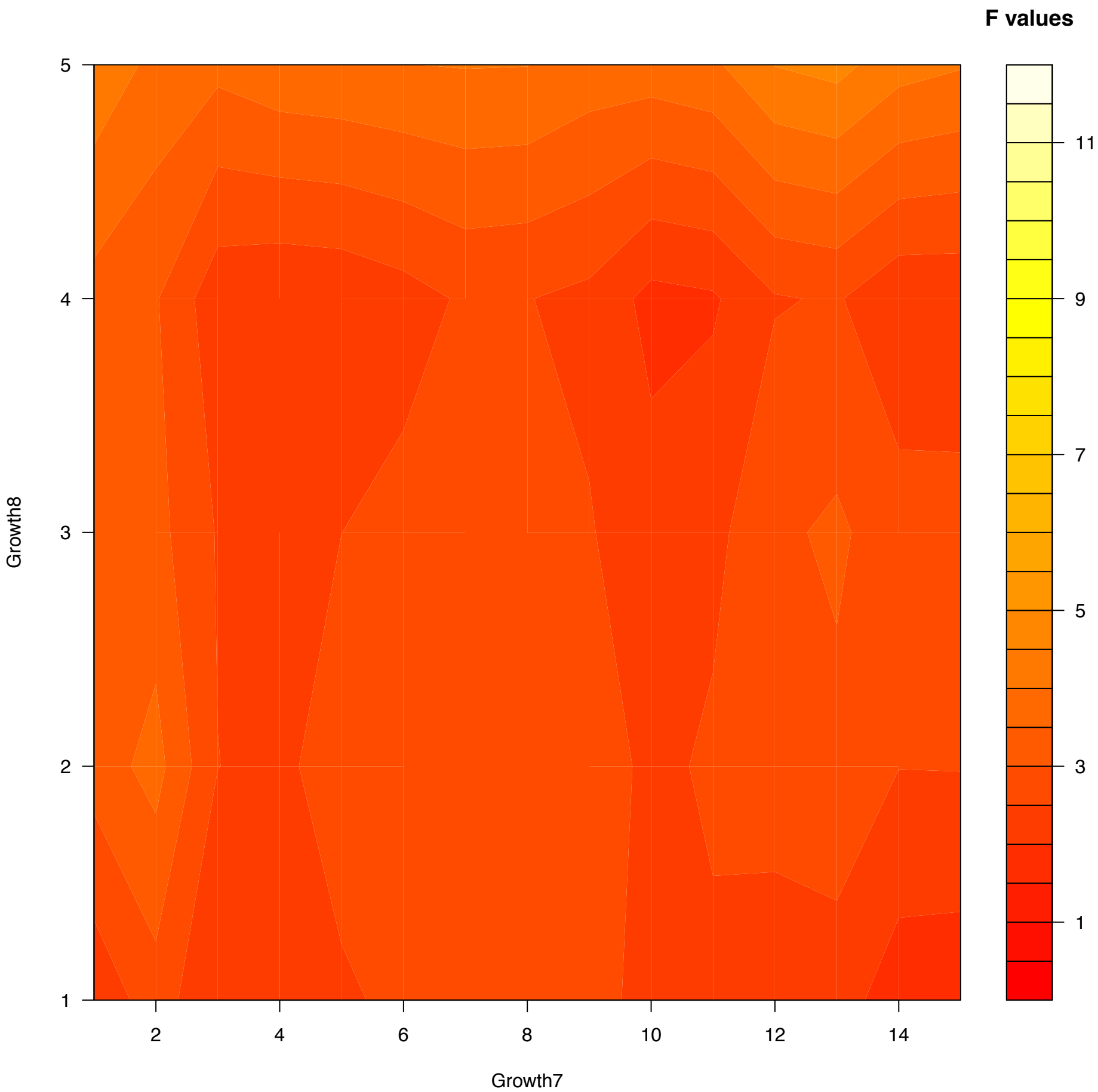

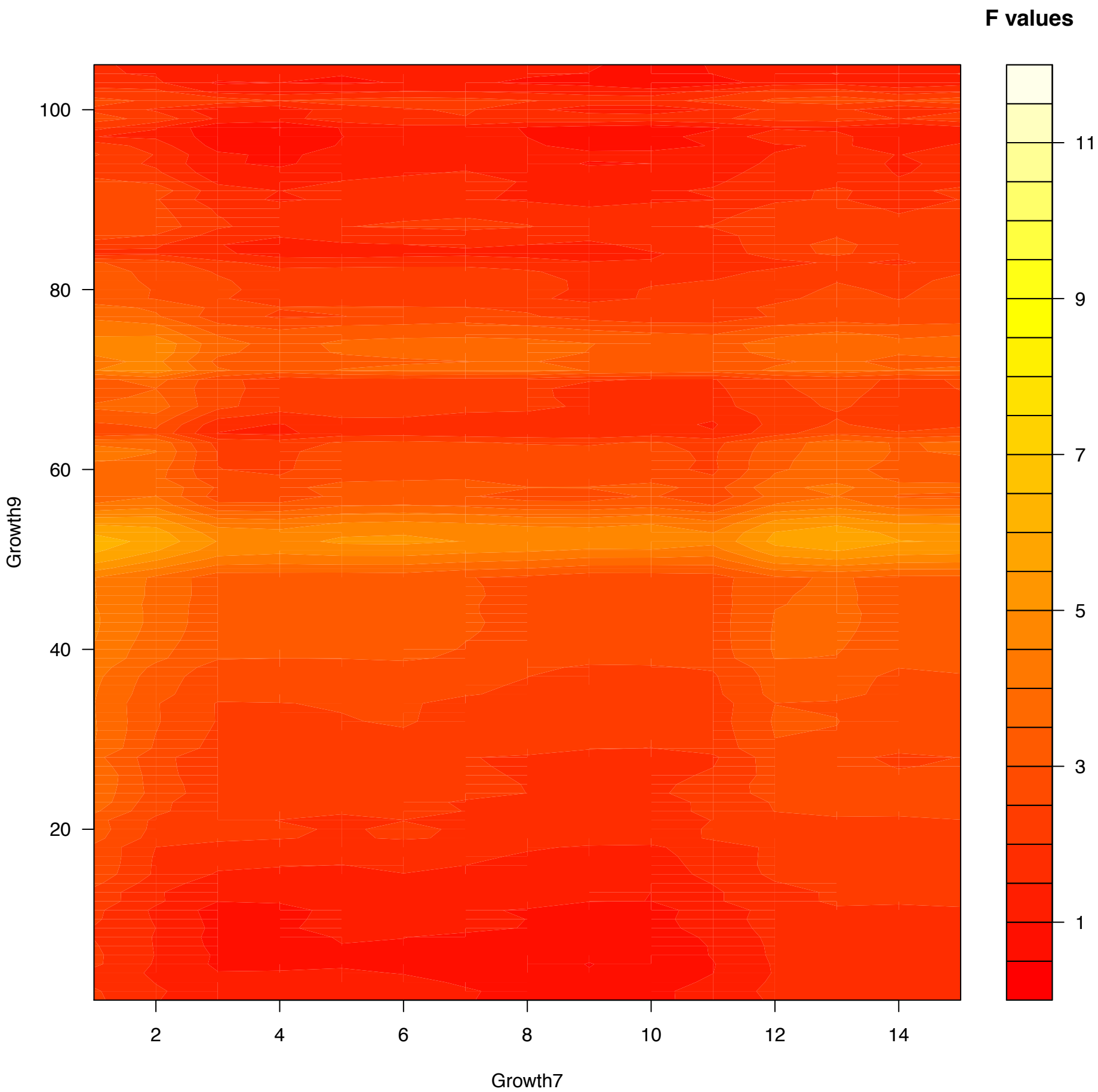

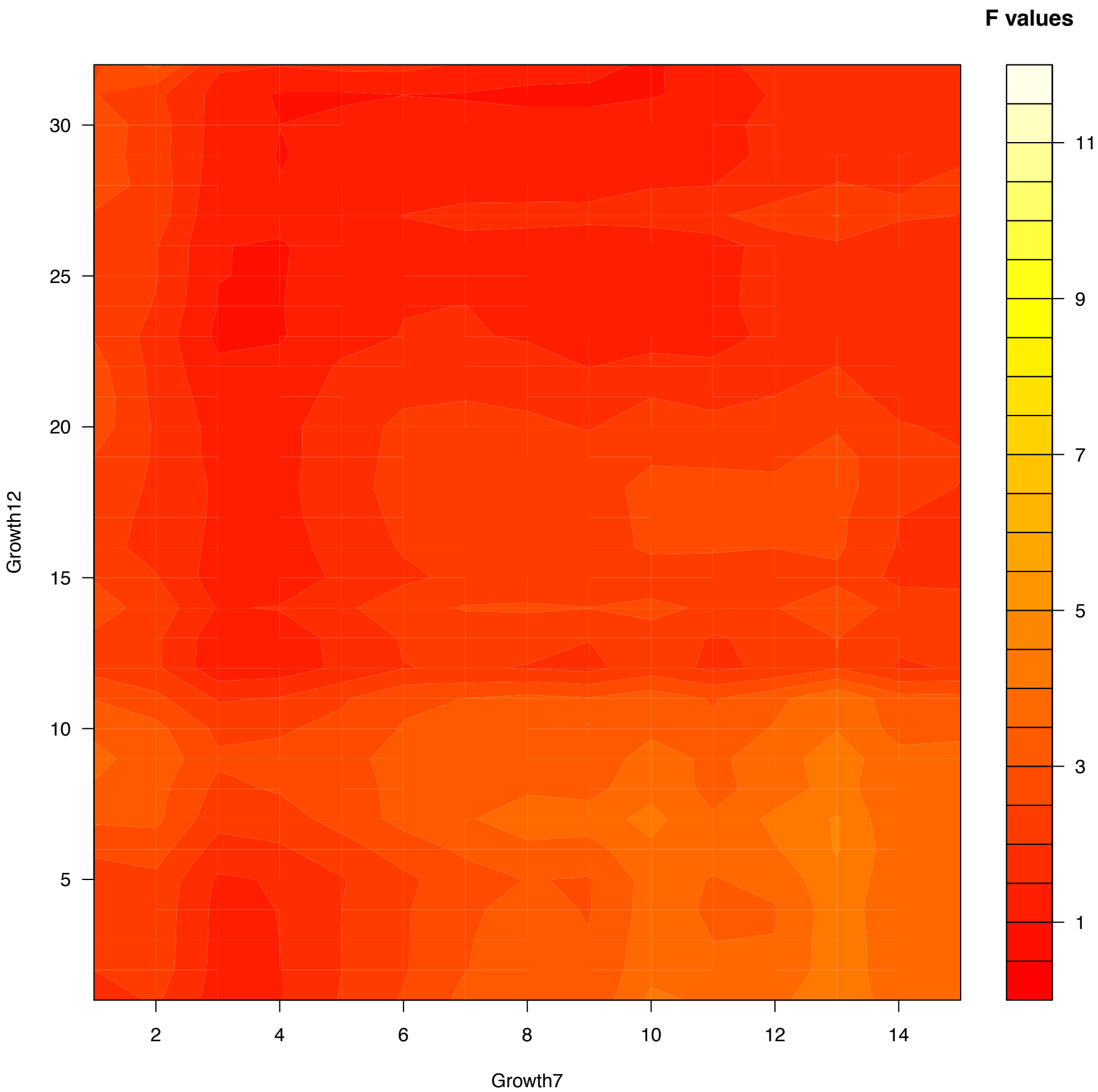

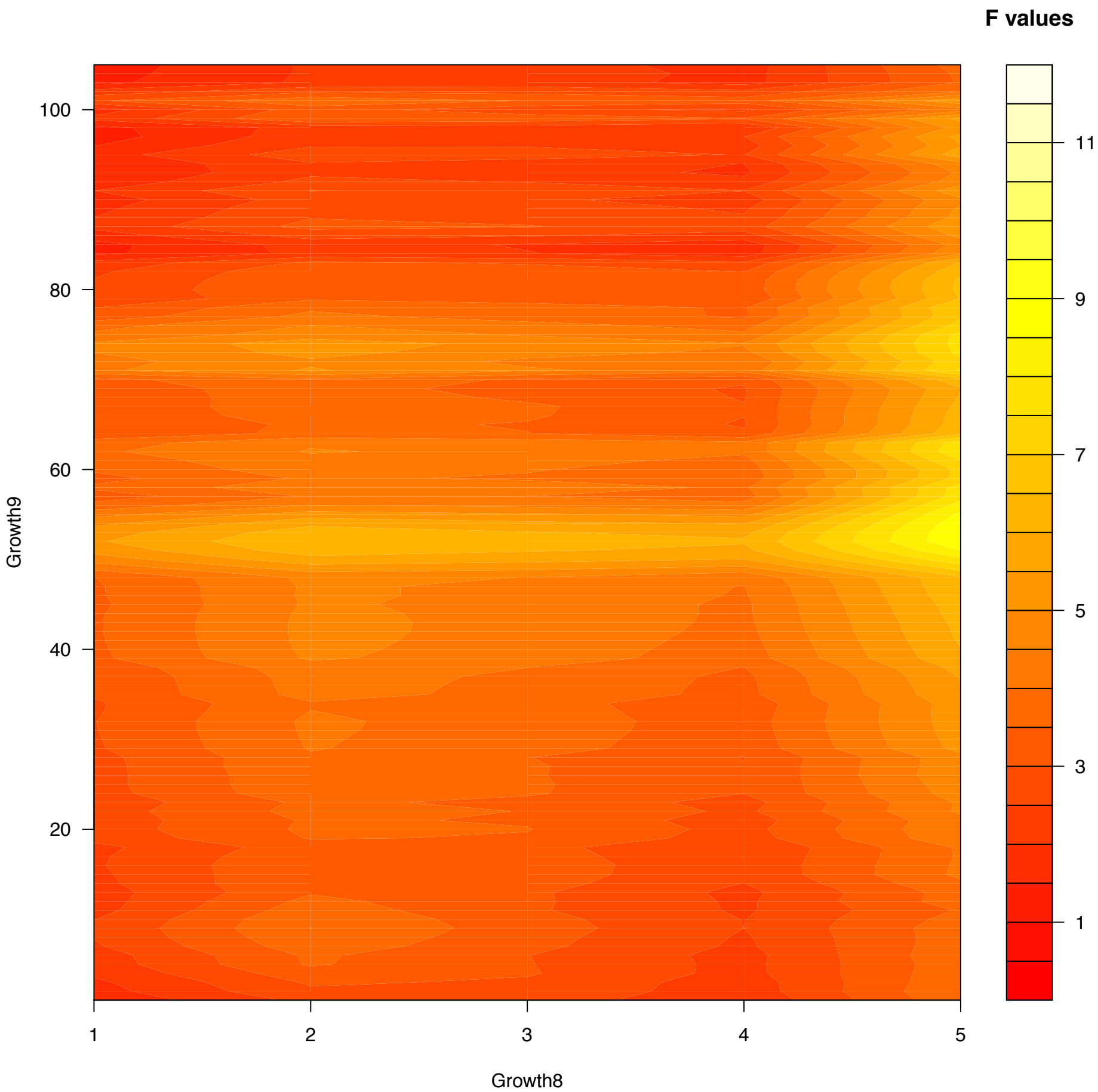

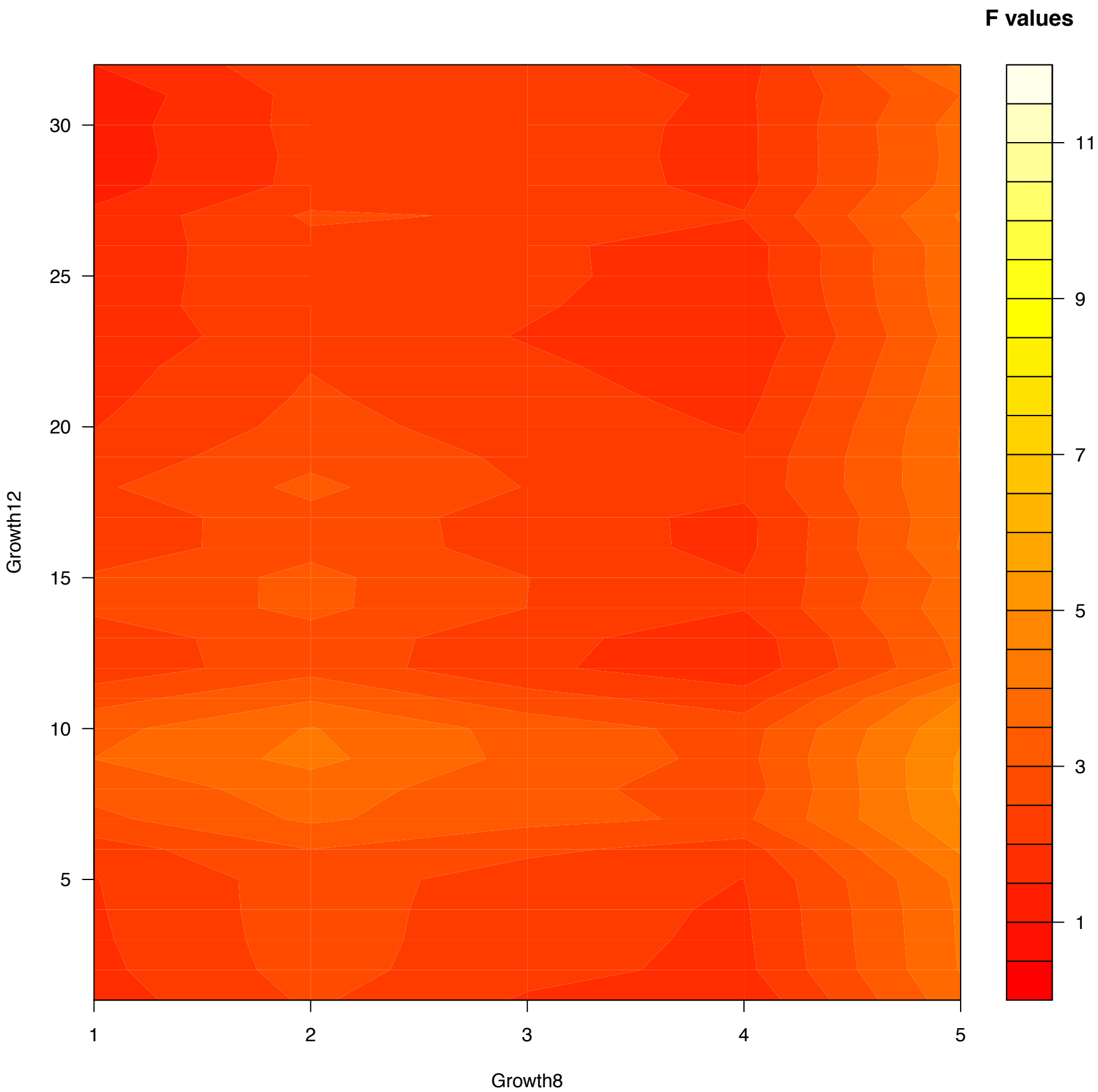

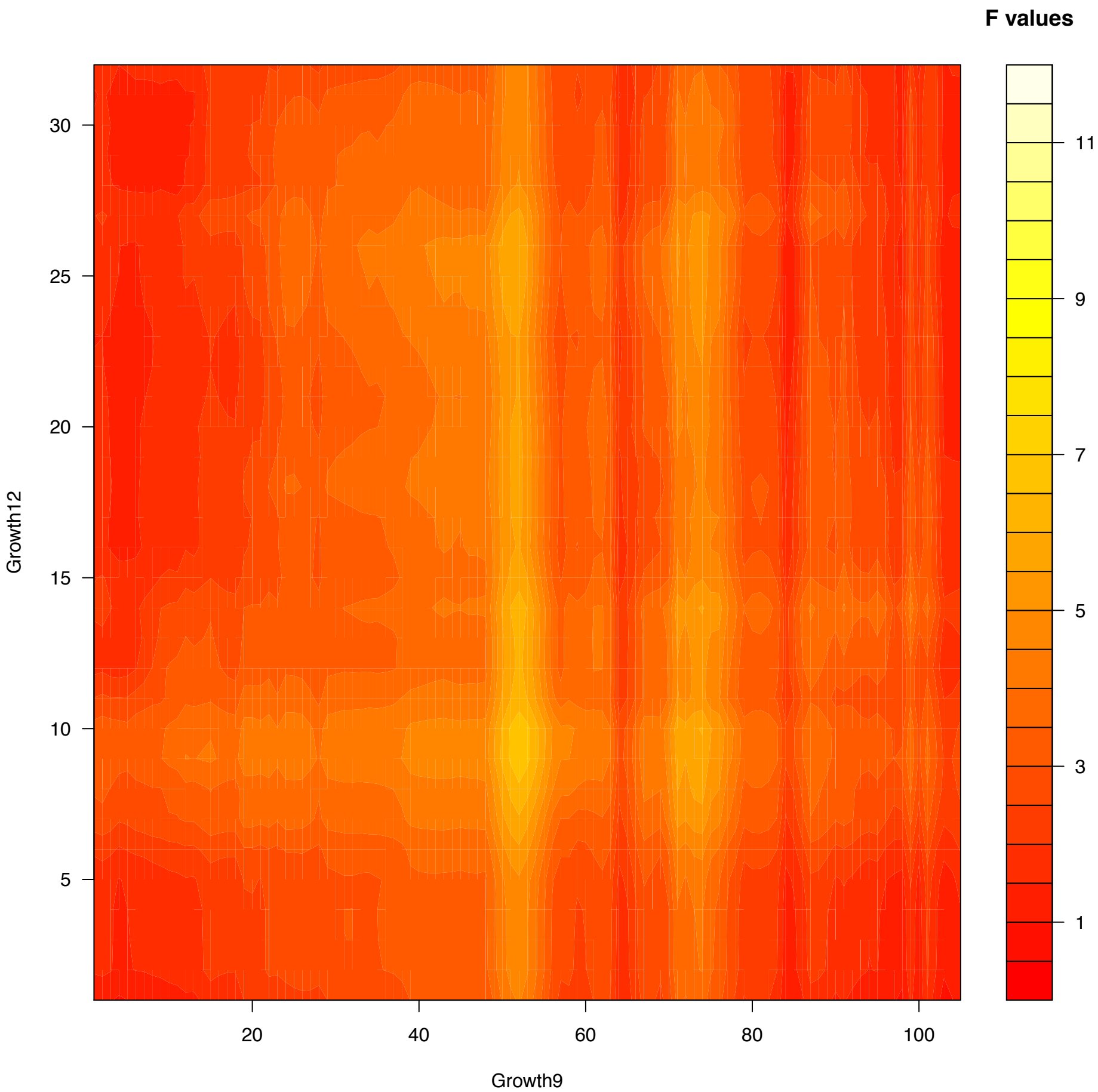

Supplement: Figure S1 — Complete 2D scan. The profile of an exhaustive 2D QTL scan over all markers in the data set. (PDF) [file pgen.1002180.s001.pdf]

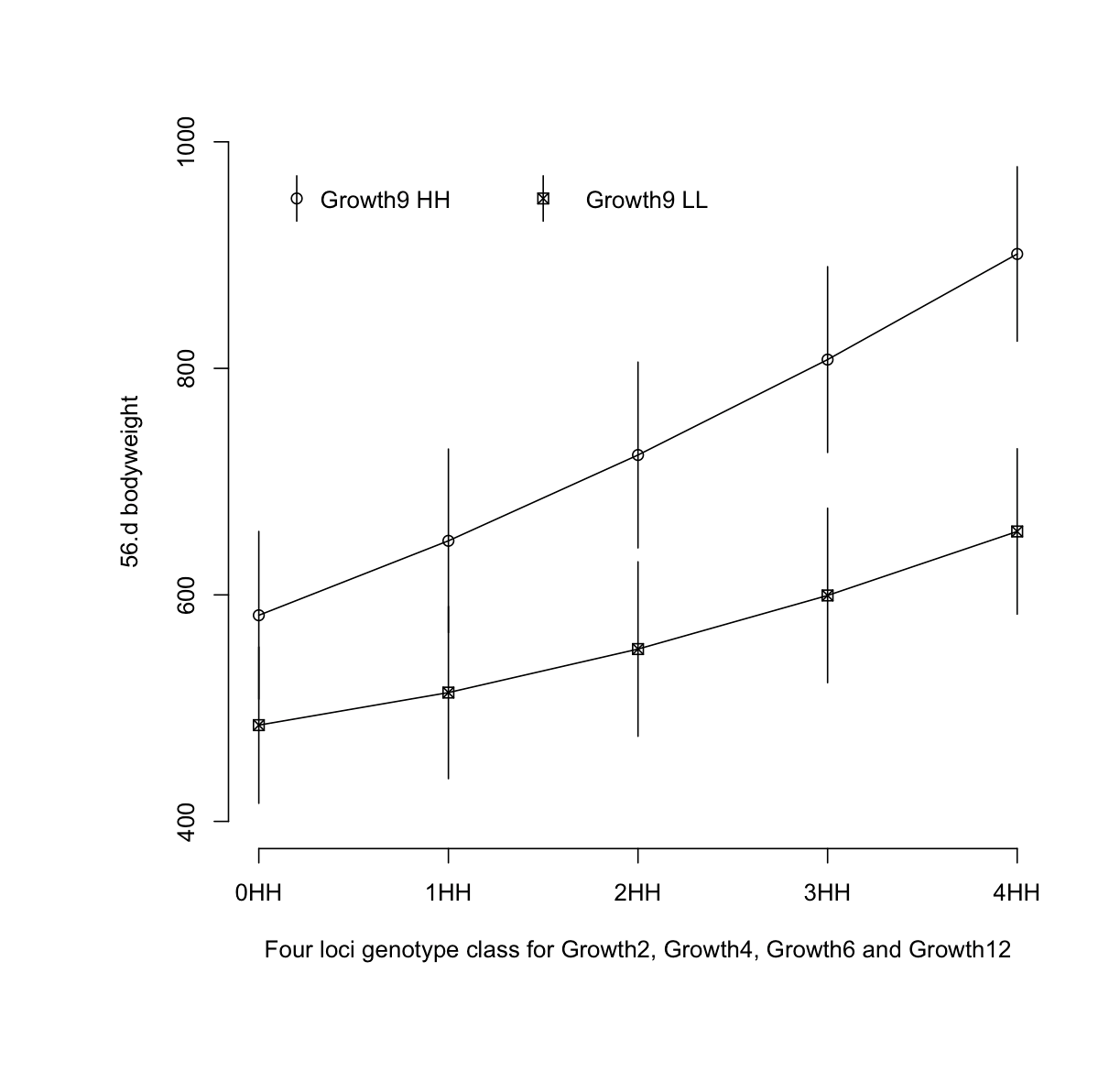

Supplement: Figure S2 — Parametric genotype-phenotype maps in HWS and LWS genetic background at Growth9.1. The phenotypic values were predicted using the NOIA model framework. The values are plotted as a function of the degree of HWS and LWS homozygosity at its interacting loci Growth2, Growth4, Growth6 and Growth12. The error bars represent s.e.m. (TIFF) [file pgen.1002180.s002.tiff]

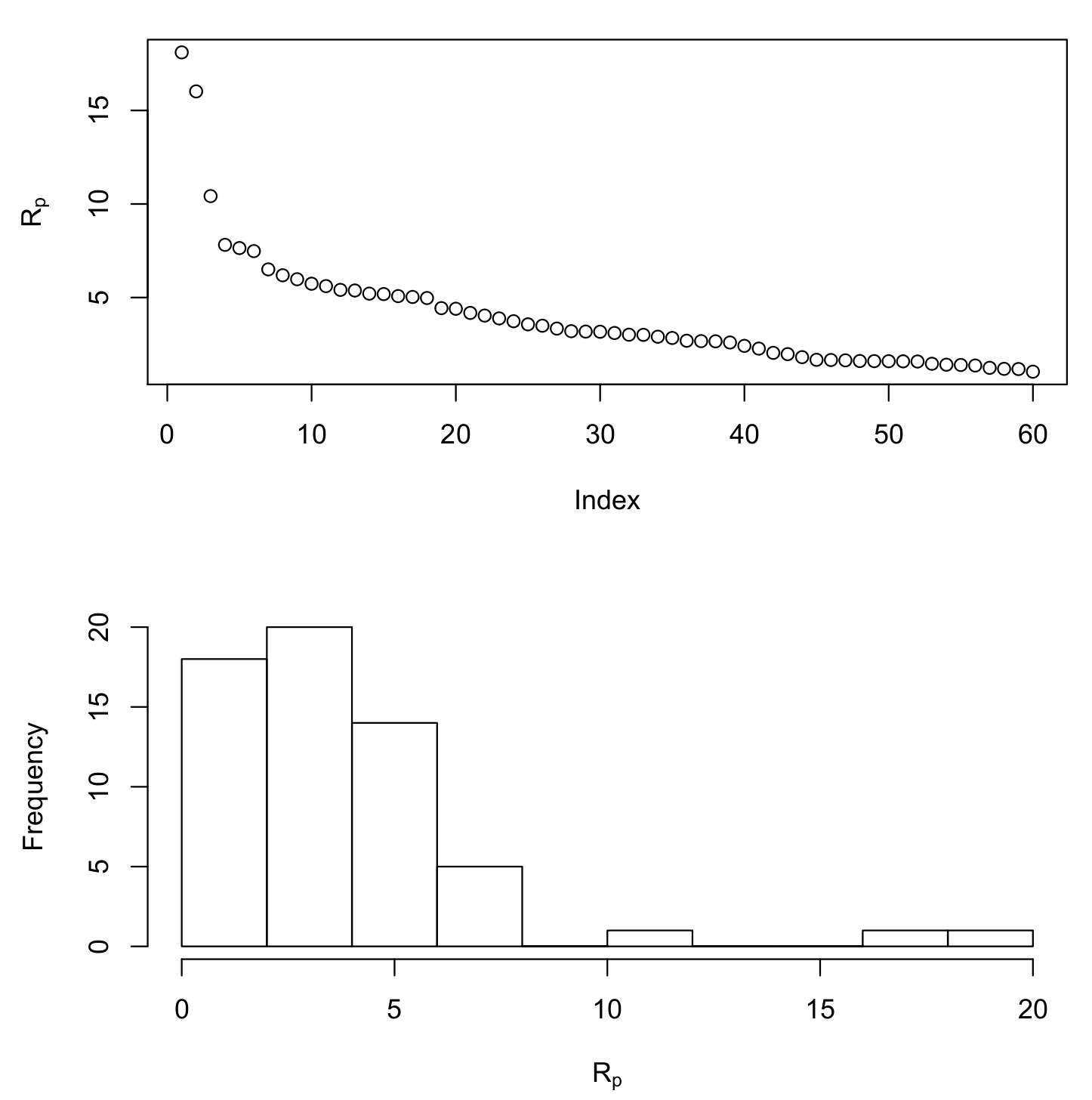

Supplement: Figure S3 — Distribution of Rp-values for all possible loci combinations. Panel a shows all values, in decreasing order, from all possible combinations within the six loci set. (60 values; 3 for each of 20 possible triplets). Panel b shows a histogram of these values. (TIFF) [file pgen.1002180.s003.tiff]
