# Supplementary material for: Replication and Explorations of High-Order Epistasis Using a Large Advanced Intercross Line Pedigree
Source: PLoS Genet. 2011 Jul 21;7(7):e1002180. doi: 10.1371/journal.pgen.1002180 (PMC3140984; doi:10.1371/journal.pgen.1002180)
Supplement: Text S1 — Prediction of multi-locus network effects using a parametric genotype-phenotype map. (DOC) [file pgen.1002180.s004.doc]

Prediction of multi-locus network effects using a parametric genotype-phenotype map

In [13], it was shown that the genetic effects of *Growth9* (including both *Growth9.1* and *Growth9.2*) depended on the number of LWS alleles in the interacting radial loci (Figure 2). Similarly, the LWS genotype at *Growth9* suppressed the genetic effects of the three interacting loci. An alternative way to the stratification based approach described above is to examine if the interaction effects in the AIL were similar to the reciprocal capacitation pattern in the F_2_ population, requiring an in-depth study of a multi-locus Genotype-Phenotype (GP) map. By estimating a five-locus GP-map for our epistatic network, we can obtain statistical predictions for the phenotype of all possible five-locus genotypes even though not all of these genotypes exist in the AIL. A convenient framework for estimating such GP-maps from QTL data is the Natural and Orthogonal InterAction (NOIA) model [18]. NOIA is a statistical framework that fits a multi-locus statistical model with epistatic effects in data where allele frequencies deviate from standard F_2_ proportions without confounding the estimates. Here, we estimated additive and dominance effects for all five loci together with all pair-wise epistatic effects of the loci (additive-by-additive, additive-by-dominance, dominance-by-additive and dominance-by-dominance) using NOIA. Using these estimates, we predicted a five-locus genotype-phenotype map for the five non-transgressive interacting loci that combined to increase the body weight in the AIL (*Growth2*, *Growth4*, *Growth6*, *Growth9* and *Growth12*). Figure S2 shows that i) the 5 locus HWS and 5 locus LWS homozygote genotypes for *Growth2*, *Growth4*, *Growth6*, *Growth9* and *Growth12* gave the highest and lowest phenotype values, respectively; ii) the expected genotypic values increased faster in the HH *Growth9* than in the LL *Growth9* genetic background.
